# A Phytogeographic Divide Along the 500 mm Isohyet in the Qinghai-Tibet Plateau: Insights From the Phylogeographic Evidence of Chinese *Allium*s (Amaryllidaceae)

**DOI:** 10.3389/fpls.2019.00149

**Published:** 2019-03-05

**Authors:** MinJie Li, DengFeng Xie, Chuan Xie, YiQi Deng, Yan Zhong, Yan Yu, XingJin He

**Affiliations:** Key Laboratory of Bio-Resources and Eco-Environment of Ministry of Education, College of Life Sciences, Sichuan University, Chengdu, China

**Keywords:** *Allium*, phytogeographic divide, genetic breaks, Pleistocene, Qinghai-Tibet Plateau

## Abstract

The Qinghai-Tibet Plateau (QTP) has been biogeographically divided into the eastern monsoonal and the western continental climatic zones along the 500 mm isohyet. However, this biogeographic hypothesis has been rarely tested using a phylogeographic approach. The members of the genus *Allium* subgenus *Cyathophora* coincidentally distribute across this biogeographical divide. Intriguingly, *Allium fasciculatum* of subgenus *Amerallium* co-occurs in the distribution range of subgenus *Cyathophora*. To illuminate the role of this biogeographic divide on the genetic divergence, we genotyped 466 individuals of 52 populations of subgenus *Cyathophora* and 110 individuals of 19 populations of *A. fasciculatum* using three chloroplast DNA fragments, whole nrITS and nine nuclear microsatellite loci, supplemented with the present environmental space and paleo-distribution modeling. Our phylogeographical evidence recovered the concordant east–west genetic breaks both for subgenus *Cyathophora* and *A*. *fasciculatum* along the 500 mm isohyet. The divergence time estimations and environmental niche differentiations suggested this east–west genetic breaks could have been triggered by the climatic-induced vicariance during the early Pleistocene. Noticeably, this split within subgenus *Cyathophora* could have been deepened by the morphological vicariance from the eastern umbel to the western spicate, while that within *A. fasciculatum* could have been obscured due to the pollen flows from the east to west caused by the postglacial expansion. The genetic structures and ecological niche modelings (ENMs) recovered the distinct responses to the Quaternary climatic oscillations for species constricted to different climatic zones, further highlighting the profound effect of the climatic differences and tectonic uplifts on the genetic diversification. Overall, our findings offer strong evidence for the existence of a biogeographic divide between the eastern monsoonal and the west continental climatic zones of the QTP nearly along the 500 mm isohyet.

## Introduction

**FIGURE 1:**
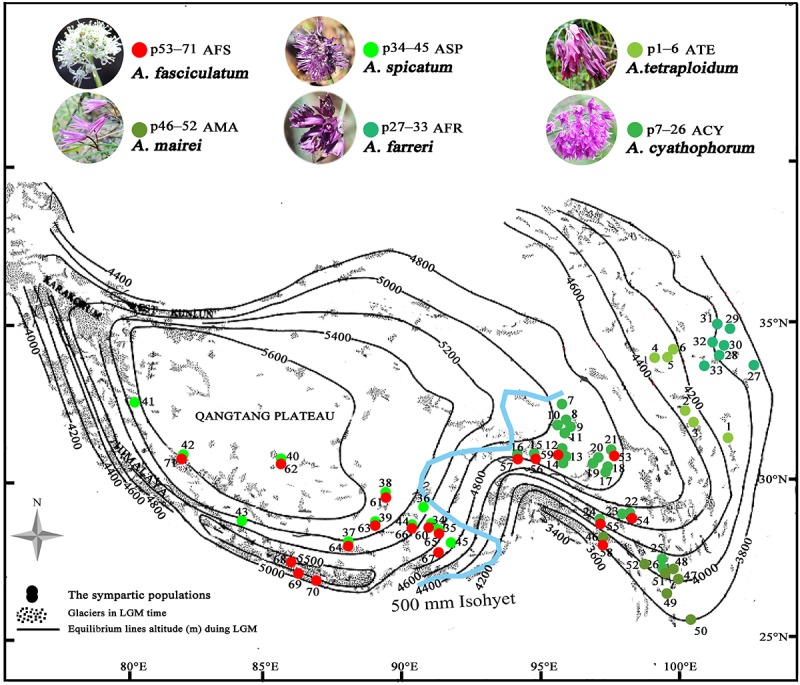
The spatial variation of the equilibrium line altitudes (ELAs) during the LGM in the QTP [referred from Shi (2002) to in honor of his contributions] and localities of sampled populations (p1–71) for subgenus *Cyathophora* species (p1–52) and *A. fasciculatum* (p53–71) across the 500 mm isohyet (blue line), which separates the QTP into the eastern monsoonal (humid–semihumid) and western continental (arid–semiarid) climatic zones [referred from Sun and Wang (2005)]. The species abbreviation holds same in the present paper: AFS, *A. fasciculatum*; AMA, *A. mairei*; ACY, *A. cyathophorum*; AFR, *A. farreri*; ATE, *A. tetraploidum*; ASP, *A. spicatum*.

The Asian monsoon system, as a by-product of the Qinghai-Tibet Plateau (QTP) uprising, has become an increasing attraction not only because of its controversial onset, also its profound effect on the reorganization of the Asian climate system (Sun and Wang, 2005; Liu and Dong, 2013). With the establishment of Asian monsoon regimes around the Oligocene/Miocene boundary, the broad arid belt which widely stretched across China from west to east in the late Cretaceous and Paleocene, has been strongly restricted to the northwest China, and the arid climate conditions have been replaced by the humid ones in East China (Sun, 1979; Guo et al., 2008; Miao et al., 2012). Undoubtedly, the episodes of the dramatic tectonic uplifts in the QTP around 15–13 Ma, 8–7 Ma and 3.5–1.6 Ma, could have strengthened the Asian monsoon at different topographic units of the QTP (Liu and Dong, 2013; An et al., 2014). This accordingly resulted in the heterogeneity of climatic conditions in the QTP (An et al., 2006, 2014; Wan et al., 2007; Zhang et al., 2012), as indicated by the occurrence of the 500 mm isohyet during these processes (Sun and Wang, 2005). It has been hypothesized that the 500 mm isohyet subdivides the QTP into the eastern monsoonal climatic zone (majorly controlled by the Indian Asian monsoon) and the western continental climatic zone (controlled by the mid-latitude westerlies). Consequently, the extreme continental glaciers with higher the actual equilibrium line altitudes (ELAs) could have developed in the western platform during the Pleistocene glaciations, whereas the dispersed mountain glaciers with lower ELAs in the eastern regions (Figure 1). This is because the ELAs determined by the balance of ablation and accumulation of glaciers, is higher in arid areas and lower in humid areas under the same temperature conditions (Shi et al., 1998; Shi, 2002; Liu and Dong, 2013). Thus, the ELAs can be used as a predictor to explore how species responded to the Quaternary climatic oscillations across the 500 mm isohyet.

The previous studies have borne out that the climatic-induced lines might have played an important role to plant divergence. For example, the ‘Tanaka-Kaiyong Line’ in Southwest China, acting as a geographic boundary of East Asian Sino-Himalayan and Sino-Japanese Floras, promoted the intraspecific divergence of *Sophora davidii* (Fan et al., 2013). Likewise, the broad arid belt now existing in the west-most part of China, worked as a climatic barrier to cause the north–south split within the Asian butternut (Bai et al., 2016). However, whether the biogeographic divide along 500 mm isohyet was also valid in the genetics and acted as a natural line of vicariant event to partition the plants in the QTP into west and east genealogies, has not been examined. Thus, pursing phylogeographic studies for the endemic plant components in the QTP regarding their genetic breaks along 500 mm isohyet during the regional monsoon intensification episodes, will shed light on the role of this biogeographic divide.

Among those endemic plant components in the QTP, the genus *Allium* L. (Amaryllidaceae) subgenus *Cyathophora* species extend their distribution range across the eastern monsoon and western continent climatic zones along the 500 mm isohyet. Strikingly, the clear morphology differences occur between populations restricted to these two zones: the eastern species comprising *A*. *mairei* (AMA), *A*. *rhynchogynum*, *A*. *cyathophorum* (ACY), *A*. *farreri* (AFR), and *A. tetraploidum* (ATE, unpublished new species, Li et al., 2016) have umbel inflorescence, while the western species only including *A*. *spicatum* (ASP) presents spicate inflorescence (Figure 1). Thus, this morphological differentiation concomitant with speciation in subgenus *Cyathophora* could be a perfect proxy to reflect the role of the biogeographic divide along 500 mm isohyet on genetic divergence. Interestingly, the field investigations found that *A. fasciculatum* (AFS) belonging to subgenus *Amerallium*, sympatrically distributes with *A. cyathophorum* in the eastern QTP, and with the *A. spicatum* in the western QTP (Figure 1 and Supplementary Table S1), however, no obvious morphology variations are presented between the east and west groups of *A. fasciculatum* (Supplementary Figure S1). In this circumstance, a comparative model can be constructed to test whether a concordant east–west genetic beak occurred both within subgenus *Cyathophora* and *A. fasciculatum* which are genetically distant. In addition, the comparisons of the genetic structures in the same/distinct climatic zone(s) can test whether the climatic differences could have an influence on the species’ responses to the Quaternary climatic oscillations. Therefore, combined with the present environmental space and the ecological niche model (ENM) of species distributions, we conducted the phylogeographical analyses for subgenus *Cyathophora* and *A. fasciculatum* using three chloroplast fragments (trnD-trnT, trnL-trnF, and rps16), whole nrITS, and nine pairs of microsatellites, aiming to (i) examine the common genetic breaks along the 500 mm isohyet; (ii) test the relevance between climatic differences and the genetic breaks; (iii) address the influence of climatic differences on species’ response to Quaternary climatic oscillations.

## Materials and Methods

### Sampling, DNA Extraction, DNA Sequencing, and Microsatellite Genotyping

Total 576 individuals were collected across 71 natural localities approximately extending the whole distribution range of *A*. *fasciculatum* and subgenus *Cyathophora*. Of which, 110 individuals for 19 populations of *A. fasciculatum*, and 466 individuals for 52 populations of all members of subgenus *Cyathophora*: 41 individuals of 7 populations of *A. mairei*, no individuals for *A*. *rhynchogynum* due to the difficulties to access, 200 individuals of 20 populations of *A. cyathophorum*, 59 individuals of 7 populations of *A. farreri*, 57 individuals of 6 populations of *A. tetraploidum*, and 109 individuals of 12 populations of *A. spicatum* (Li et al., 2016) (Supplementary Table S1). *Allium macranthum* was used as outgroup to examine the monophyly of *A. fasciculatum* (Supplementary Figure S2). Voucher specimens were deposited in Sichuan University Herbarium (SZ).

Total genomic DNA was isolated from the well silica-dried foliar tissue using a protocol of plant genomic DNA kit (Tiangen Biotech, Beijing, China). Three chloroplast DNA fragments (trnD^GUC^-trnT^GGU^, trnL^UAA^-trnF^GAA^, and rps16) and the whole nrITS were amplified using the common primer pairs for the individuals of *A. mairei* and *A. fasciculatum*. One individual of *Allium macranthum* was amplified to confirm the monophyly of *A. fasciculatum* in the cpDNA-based phylogenetic analyses. For trnD^GUC^-trnT^GGU^ and trnL^UAA^-trnF^GAA^, the PCR began with 94°C for 4 min, followed by 35 cycles of 94°C for 45 s, 60°C for 1 min, and 72°C for 1 min with a final-extension for 10 min at 72°C. For rps16 and nrITS, the most conditions followed the above one, except the different denaturation temperatures, respectively, at 52°C and 55°C (Supplementary Table S2). PCR for all primers was conducted in a 30 μL volume comprising 3 μL plant total DNA, 0.8 μL forward primer, 0.8 μL reverse primer and 15 μL 2× Taq MasterMix (cwbio, Beijing, China). The purified PCR products of DNA sequences were bidirectionally sequenced with the primers in ABI Genetic Analyzer (Applied Biosystems Inc., Foster City, CA, United States). Using a double-suppression PCR method, nine pairs of microsatellite primers from 25 ones (Lee et al., 2011; Hyoun-Joung et al., 2012) (Supplementary Table S2), were cross-amplified and genotyped for these 576 individuals in this study. PCR products were separated on 3.5% of agarose gel followed by staining with ethidium bromide. Alleles were sized using Peak Scanner software v.1.0 (Applied Biosystems).

### Genetic Dataset Analysis

DNA sequence was edited to get consensus sequence using SeqMan (DNAstar, Burland, 2000). Sequence alignment was conducted in Clustal X version 2.0 (Larkin et al., 2007), followed by fine manual adjustment. DNAsp v 5.0 (Librado and Rozas, 2009) was used to generate the haplotype files and to estimate haplotype diversity (Hd) and nucleotide diversity (π). Genealogical relationships among haplotypes were inferred from a median-joining method optimized by maximum parsimony in Network 4.6.1.3^1^. The neutrality tests for DNA sequences using the Tajima’s *D* test and Fu’ Fs statistics were calculated by Arlequin version 3.5 (Excoffier and Lischer, 2010). The newly haplotype sequences were submitted to GenBank with KY744819–KY744939. The haplotypes of DNA sequences for *A. cyathophorum*, *A. farreri*, *A. tetraploidum*, and *A. spicatum* were cited from Li et al. (2016) with GenBank numbers KP164313–KP164411, KP114563–KP114599, KP127671–KP127673, and KT369742–KT369758.

MICRO-CHECKER v. 2.2.3 (Van et al., 2004) and Cervus v. 3 (Marshall et al., 2010) were used to test for evidence of deviations from the Hardy–Weinberg equilibrium, genotyping errors and null alleles. FSTAT 2.9.3 (Goudet, 2001) was used to estimate the genetic diversity statistics of nSSRs dataset. For each microsatellite locus, the genetic diversity was estimated using the observed number of alleles (A_O_), the observed heterozygosity (H_O_), the expected heterozygosity (H_E_), the average genetic diversity within the population (H_S_) and fixation index F_is_. For each population, the genetic diversity was assessed across all loci using A_O_, H_O_, H_S_, F_is_, and allele richness (R_S_). Linkage disequilibrium (LD) among loci was tested in FSTAT using Delta’ and *R*^2^. Genetic groups in the nSSR dataset were identified by a Bayesian model-based clustering approach using STRUCTURE v2.3.4 (Pritchard et al., 2010), following the admixture model and assuming independent allele frequencies among populations. Ten independent runs were performed for each *K* (=1–20) with 100,000 MCMC replications and 10,000 used as burn-in steps. To determine the true number of gene pools indicated by the optimal *K*-value, we monitored the mean of log_e_P(D), with independent runs for each K (Pritchard et al., 2000). Alternatively, plots of *ad hoc* posterior probability models of Delta K were also observed to predict the most appropriate K (Evanno et al., 2005). Principal coordinate analysis (PCoA) was conducted on the microsatellite data using GENALEX 6.5 (Peakall and Smouse, 2012).

Using DNA sequences, the phylogeographic structure for each taxon was estimated in PermutCpSSR1.2.1 (Pons and Petit, 1996) by calculating interpopulation differentiation (G_ST_) and the number of population subdivision (N_ST_), with running 1,000 random permutations for the significance of N_ST_ > G_ST_. The average genetic diversity within populations (H_S_), total genetic diversity (H_T_) were also computed. An analysis of molecular variance (AMOVA) was conducted using ARLEQUIN version 3.5 to quantify variation in DNA sequences (cpDNA and nrITS) and nSSRs using Φ- and R- statistics, with 10,000 permutations to test the significance (Excoffier et al., 1992).

### The Divergence Time Estimations

In the absence of known palaeo-botanical information for *Allium* or related genera to calibrate tree nodes, a constant mutation rate 1.52 × 10^-9^ s/s/y within the cpDNA dataset (Yamane et al., 2006) was used to estimate the divergence time in BEAST v.2.5.0 (Bouckaert et al., 2014). The best-fitting substitution model (GTR+I+G) was calculated in jModelTest 2.1.10 (Posada, 2008). An uncorrelated lognormal relaxed clock and Yule process tree prior was specified with a random starting tree. A total of 50,000,000 generations are set in Markov chain, both echo state to screen and log parameters every 5,000 generations. The first 10% trees were discarded as burin-in and the remaining were used to get a maximum clade credibility (MCC) tree with TreeAnnotator v1.10. Finally, MCC tree with mean ages for each node and their 95% credible intervals was displayed in Fig Tree 1.4.3^2^.

### Mantel Correlations for Diverged Lineages to Environment and Geographical Distances (i.e., Isolation by Environment or Distance, IBE or IBD)

To test whether there was a correlation between speciation and climatic factors, ecological niche divergence among the taxon was estimated. The distribution information of each species was tabulated in Supplementary Table S3, including 9 georeferenced occurrence records for *A. tetraploidum*, 25 for *A. cyathophorum*, 13 for *A. farreri*, 23 for *A. spicatum*, 33 for *A. mairei*, and 52 for *A. fasciculatum*. We retrieved 19 current bioclimatic variables at 30 arc-seconds (Bio1–Bio19; Supplementary Table S4) and altitude from WorldClim website^3^ (Hijmans et al., 2005) for each georeferenced location. The determinant bioclimatic variables which exhibited pairwise Pearson correlation coefficients *r* < 0.7 for each taxon of interest were finally selected. Of which, Bio1 Annual mean temperature, Bio2 Mean diurnal range, Bio3 Isothermality, Bio4 Temperature seasonality, Bio12 Annual precipitation, Bio14 Precipitation of driest month, Bio15 Precipitation seasonality, Bio17 Precipitation of driest quarter and Altitude were included (Supplementary Table S5). A principle component analysis (PCA) was first performed to identify the ecological niche differentiation based on the determined bioclimatic variables using *prcomp* function (Dray and Dufour, 2007). The PCA loading scores for axes one (PC1) and two (PC2) of each determined bioclimatic variable were extracted using *get_pca_ind* function in R factoextra package. ANOVA permutation analysis (PERMANOVA) was then conducted to estimate the variation of principle components (PC1 and PC2) and each determinant bioclimatic variable within the taxa of interest using R lmPerm package to identify the bioclimatic variables which determined species’ divergence (Wheeler, 2010). The principle components and bioclimatic variables with significant differences indicated by the ANOVA were further confirmed by the Tukey’s honestly significant difference (HSD) test using the R Stats package.

To assess the effect of geographic distances and environmental conditions (i.e., IBD and IBE) to the observed genetic differentiation, the correlations between pairwise F_ST_ estimated from the microsatellite markers and geographic distance were investigated, as well as between pair F_ST_ and environmental distance. A matrix of pairwise F_ST_ between populations was calculated using ARLEQUIN version 3.5. A matrix of geographic distance was calculated by the geographic coordinates (Supplementary Table S1) using GENALEX 6.5, and matrices of the environmental distance were computed using the significantly different bioclimatic variables, PC1 and PC2 on a basis of Euclid method. Mantel tests were conducted on the matrices of pairwise F_ST_/(1–F_ST_) and geographical/environmental distances by running 10,000 permutations in R vegan package.

### The Ecological Niche Modeling

The ecological niche modeling (ENM) was performed in MAXENT v3.3.1 (Phillips and Dudik, 2008) to predict potential distribution range shifts for involved species at the present, the Last Glacial Maximum [LGM, *c*. 21 kyr before present (BP)] and the Last inter-glacial (LIG, *c*. 130 kyr BP), respectively. These determinant bioclimatic variables exhibiting pairwise Pearson correlation coefficients *r* < 0.7 of each group (Supplementary Table S5) were used to perform ENM for current distributions. The ecological layers for the last glacial maximum (LGM) and Last Inter-Glacial were also retrieved from the WorldClim website at 2.5 arc-min and 30 arc-seconds spatial resolution, respectively. For LGM prediction, we used data from the Model for Interdisciplinary Research on Climate (MIROC). 75% of the distribution sites were randomly selected as a prediction program with the commission error taken into consideration, and the remaining were conducted to estimate the goodness of the prediction. The maximum number of iterations and the convergence threshold were set at 1,000 and 1 × 10^-5^, respectively. The accuracy of each model prediction was evaluated by using the area under the receiver operating characteristic curve (AUC; Fawcett, 2006).

## Results

### cpDNA Diversity and Network Topology

The alignment of three concatenated plastid markers (trnD-trnT, trnL-trnF and rps16) has a consensus length of 2,249 bp and 2,242 bp, respectively, for subgenus *Cyathophora* and *A*. *fasciculatum*, with 46 and 24 chlorotypes (Table 1) based on 91 (Supplementary Table S6) and 42 variable sites (Supplementary Table S7). It was found that the species including *A. mairei* (H_T_/H_S_ = 1.0/0.137), *A. farreri* (H_T_/H_S_ = 0.847/0.303), and *A. fasciculatum* (H_T_/H_S_ = 0.913/0.408) showed much higher H_T_ than H_S_, and concordantly have the significant phylogeographic structures (N_ST_ > G_ST_, all *P* < 0.05). The similar genetic structures were also detected in the east (H_T_/H_S_ = 0.539/0.287) and west groups (H_T_/H_S_ = 0.921/0.479) of *A. fasciculatum*. By contrast, the species with no such significant differences between H_T_ and H_S_, presented the non-significant phylogeographic structures (N_ST_ > G_ST_, both *P* > 0.05), i.e., in *A*. *spicatum* (H_T_/H_S_ = 0.789/0.541) and *A. tetraploidum* (H_T_/H_S_ = 0.257/0.170), and even no such phylogeographic structure (N_ST_ = 0.344, G_ST_ = 0.539, *P* > 0.05) in *A*. *cyathophorum* (H_T_/H_S_ = 0.826/0.381) (Table 2). The nucleotide diversity (π) of western genealogies (both *A*. *spicatum* and the west group of *A. fasciculatum*, 0.50 × 10^-3^ and 0.93 × 10^-3^) was greatly larger than that of the eastern genealogies (ranging from 0.03 × 10^-3^ to 0.24 × 10^-3^) (Table 2). Neutrality test statistics based on Tajima’s *D* and Fu’s Fs were generally non-significant for all taxa (Supplementary Table S8).

**Table 1:** Geographic and genetic characteristics of 52 populations of subgenus *Cyathophora* species (AMA, ACY, AFR, ATE, ASP) and 19 populations of *A*. *fasciculatum* (AFS) based on the variability of DNA sequences (cpDNA and nrITS) and nuclear microsatellites (nSSRs).

Species	Code	Microsatellite					cpDNA	nrITS
				
		A_O_	H_O_ (SE)	H_S_	R_S_	F_is_	Chlorotypes	Ribotypes
**ATE**	LH1	14	1.000 (0.000)	0.067	2.2	-0.355	C4(7)	R1(7)
	BM2	25	0.936 (0.064)	0.310	1.9	0.207	C1(10)	R1(10)
	MR3	22	0.927 (0.073)	0.256	2.0	-0.138	C1(10)	R1(5), R2(5)
	JR4	24	0.927 (0.063)	0.296	1.6	0.109	C1(9), C2(1)	R1(7), R3(3)
	LJ5	19	0.918 (0.082)	0.179	1.5	0.189	C1(9), C3(1)	R1(10)
	HB6	18	0.927 (0.073)	0.148	1.3	-0.102	C1(10)	R1(10)
**ACY**	XSM7	19	0.211 (0.087)	0.248	1.7	0.149	C5(10)	R4(7), R5(3)
	JXF8	16	0.111 (0.056)	0.181	1.5	0.388	C5(3), C6(6), C7(1)	R4(5), R5(5)
	NQ9	16	0.144 (0.073)	0.176	1.5	0.182	C7(2), C8(3), C9(1), C10(2), C11(2)	R4(10)
	MZ10	18	0.178 (0.097)	0.232	1.4	-0.056	C5(10)	R4(5), R5(5)
	KD11	14	0.333 (0.150)	0.141	1.6	-0.579	C5(10)	R4(8), R5(2)
	XL12	19	0.222 (0.138)	0.240	1.6	0.211	C12(9), C13(1)	R6(10)
	LW13	16	0.178 (0.060)	0.248	1.6	0.284	C13(8), C14(2)	R5(1), R6(7), R7(1), R8(1), R9(2)
	KM14	16	0.178 (0.060)	0.246	1.5	-0.173	C6(10)	R4(2), R5(17), R9(2), R10(1), R11(1)
	DQ15	16	0.289 (0.116)	0.190	1.6	-0.055	C13(10)	R5(10)
	SX16	18	0.211 (0.099)	0.230	2.1	-0.110	C13(10)	R5(10)
	XP17	26	0.267 (0.104)	0.400	2.4	-0.028	C6(2), C15(8)	R5(1), R6(7), R12(1)
	GJ18	30	0.400 (0.107)	0.497	1.9	-0.006	C15(10)	R6(4), R13(2), R14(2), R15(1)
	CD19	22	0.456 (0.119)	0.359	1.9	-0.146	C5(10)	R5(10)
	JY20	24	0.444 (0.129)	0.331	1.8	-0.209	C5(8), C8(1), C14(1)	R5(3), R6(1), R12(6), R13(1), R16(2), R17(1), R18(1), R19(1)
	GT21	23	0.411 (0.121)	0.294	2.1	-0.396	C13(10)	R1(9), R20(1)
	ZB22	25	0.378 (0.124)	0.397	2.0	0.104	C6(1), C16(9)	R6(10)
	MK23	30	0.383 (0.103)	0.400	1.9	-0.041	C6(6), C17(14)	R6(20)
	ZG24	26	0.434 (0.117)	0.354	1.9	0.173	C2(1), C6(1), C13(6), C17(2)	R6(8), R13(2), R21(1)
	WF25	20	0.315 (0.081)	0.357	1.9	-0.140	C6(1), C17(6)	R6(1), R22(4), R23(1)
	YZ26	15	0.296 (0.103)	0.259	1.7	0.429	C17(3)	R6(1), R22(2)
**AFR**	ZQ27	20	0.244 (0.104)	0.290	1.7	0.156	C18(9)	R24(7)
	DB28	13	0.370 (0.161)	0.204	1.7	-0.818	C22(1)	R24(1)
	KL29	19	0.284 (0.128)	0.220	1.6	-0.291	C19(6), C21(4)	R29(10)
	ZN30	15	0.256 (0.117)	0.223	1.6	-0.144	C23(7), C24(2)	R24(5), R25(1), R29(1)
	XH31	15	0.352 (0.129)	0.233	1.5	-0.508	C19(4), C21(2)	R24(1), R25(1), R29(3), R30(1)
	LQ32	19	0.367 (0.126)	0.341	1.5	-0.076	C19(7), C20(3)	R24(4), R25(5)
	MQ33	16	0.300 (0.151)	0.284	1.4	-0.057	C19(9), C20(1)	R25(6), R26(1), R27(1), R28(1)
**ASP**	LS34	25	0.289 (0.102)	0.483	2.2	0.402	C27(1), C28(8), C30(1)	R31(8), R32(1), R33(2), R34(1)
	ND35	22	0.333 (0.118)	0.403	2.0	0.172	C25(2), C26(1), C27(2), C28(3), C33(1)	R31(5), R32(5)
	LZX36	14	0.222 (0.097)	0.283	1.6	0.216	C26(2), C28(1), C35(1), C36(1)	R31(2), R32(1)
	LZ37	13	0.404 (0.122)	0.222	2.4	-1.000	C34(1)	R32(1)
	NM38	32	0.444 (0.176)	0.491	1.4	0.177	C25(10), C26(2), C27(1)	R31(1), R32(12)
	RK39	15	0.325 (0.089)	0.272	2.0	0.184	C25(2), C26(1), C27(3)	R31(3), R32(5), R36(1)
	CQ40	29	0.289 (0.087)	0.492	1.5	0.413	C25(5), C26(1), C27(1), C28(1), C31(1), C32(1)	R31(2), R32(11), R35(3)
	GE41	24	0.389 (0.123)	0.408	2.2	0.045	C25(10)	R31(1), R32(2), R35(1)
	PL42	27	0.433 (0.114)	0.471	2.3	0.081	C25(8), C26(2)	R31(2), R32(2), R35(1)
	ZB43	30	0.389 (0.092)	0.488	2.3	0.204	C25(2), C26(4), C27(4), C28(1)	R31(3), R32(7)
	LKZ44	21	0.344 (0.113)	0.362	1.9	0.048	C25(2), C26(2), C27(2), C28(5), C29(1)	R31(3), R32(8)
	ML45	26	0.344 (0.093)	0.407	2.1	0.153	C25(7), C26(2), C28(1)	R32(10)
**AMA**	CY46	19	0.389 (0.100)	0.093	1.2	-0.066	C46(5)	R38(5)
	LJ47	11	0.148 (0.113)	0.324	1.7	-0.0600	C43(3)	R37(3)
	HB48	15	0.306 (0.137)	0.378	1.8	0.057	C37(4)	R45(4)
	JC49	17	0.244 (0.099)	0.370	1.8	0.353	C44(4), C45(1)	R39(4), R44(1)
	CS50	17	0.278 (0.106)	0.275	1.7	0.250	C42(4)	R46(4)
	ZD51	20	0.189 (0.081)	0.504	2.4	0.312	C40(1), C41(9)	R39(6), R40(4)
	DQ52	29	0.389 (0.103)	0.365	1.9	0.228	C38(8), C39(2)	R41(4), R42(5), R43(1)
**AFS**	GT53	21	0.432 (0.153)	0.342	1.8	-0.264	C56(4)	R47(3), R49(1)
	ZB54	20	0.370 (0.132)	0.323	1.8	-0.146	C56(6), C58(2), C59(1)	R47(9)
	ZG55	21	0.398 (0.146)	0.327	1.8	-0.218	C56(8), C67(2)	R47(10)
	DQ56	20	0.400 (0.135)	0.327	1.8	-0.223	C56(6), C57(1), C58(3)	R47(8), R49(2)
	SX57	15	0.417 (0.144)	0.296	1.7	-0.406	C56(3), C57(1)	R47(3), R49(1)
	CY58	12	0.333 (0.167)	0.167	1.3	-1.000	C56(3)	R47(3)
	LWQ59	12	0.333 (0.167)	0.167	1.3	-1.000	C68(3)	R51(3)
	LS60	13	0.333 (0.167)	0.185	1.4	-0.800	C69(3)	R52(3)
	NML61	12	0.306 (0.155)	0.167	1.3	-0.833	C48(1), C50(1), C51(2)	R47(2), R48(2)
	CQ62	20	0.556 (0.149)	0.426	2.0	-0.304	C48(3), C52(1)	R48(4)
	RK63	15	0.593 (0.165)	0.315	1.7	-0.882	C70(3)	R47(3)
	LZ64	22	0.556 (0.126)	0.439	2.0	-0.266	C48(2), C60(6), C61(1), C62(1)	R48(10)
	ND65	17	0.422 (0.122)	0.303	1.7	-0.394	C48(2), C64(1), C65(2)	R47(5)
	LKZ66	30	0.519 (0.120)	0.437	2.2	-0.187	C48(5), C50(1), C52(1), C53(1), C54(2)	R47(10)
	CM67	28	0.511 (0.120)	0.474	2.3	-0.078	C52(5), C55(6)	R47(11)
	JL68	16	0.444 (0.136)	0.352	1.8	-0.263	C50(2), C66(1)	R48(3)
	NLM69	19	0.460 (0.125)	0.380	1.9	-0.213	C63(7)	R48(7)
	QM70	15	0.417 (0.150)	0.329	1.7	-0.268	C48(4)	R48(4)
	PL71	15	0.333 (0.167)	0.167	1.3	-1.000	C47(1), C48(1), C49(1)	R48(2), R50(1)


*Species abbreviations following Figure 1: *A. fasciculatum*, AFS; *A. mairei*, AMA; *A. cyathophorum*, ACY; *A. farreri*, AFR; *A. tetraploidum*, ATE; *A. spicatum*, ASP. A_*O*_, the observed number of alleles; H_*O*_, the observed heterozygosity; H_E_, the expected heterozygosity; H_*S*_, the average genetic diversity within the population; Fis, fixation index; R_*S*_, allele richness.*

**Table 2:** Estimates of the nucleotide diversity (π), average genetic diversity within populations (H_S_), total genetic diversity (H_T_), interpopulation differentiation (G_ST_), and the number of population subdivision (N_ST_) (mean ± SE in parentheses) on cpDNA and nrITS datasets for subgenus *Cyathophora* species and *A. fasciculatum*.

Taxon	π × 10^-3^ (±*SD*)	H_S_ (±*SD*)	H_T_ (±*SD*)	G_ST_ (±*SD*)	N_ST_ (±*SD*)
**cpDNA**					
AFS	0.64 (±0.99)	0.408 (±0.080)	0.913 (±0.034)	0.553 (±0.088)	0.756 (±0.060)^∗^
West-AFS	0.93 (±1.16)	0.479 (±0.109)	0.921 (±0.040)	0.480 (±0.126)	0.714 (±0.137)^∗^
East-AFS	0.14 (±0.14)	0.287 (±0.106)	0.539 (±0.140)	0.467 (±0.258)	0.788 (±0.166)^∗^
AMA	0.09 (±0.13)	0.137 (±0.068)	1.000 (±0.017)	0.863 (±0.068)	0.985 (±0.011)^∗^
ACY	0.24 (±0.39)	0.381 (±0.070)	0.826 (±0.035)	0.539 (±0.076)	0.344 (±0.058)
AFR	0.14 (±0.11)	0.303 (±0.089)	0.847 (±0.081)	0.642 (±0.140)	0.821 (±0.100)^∗^
ATE	0.03 (±0.05)	0.170 (±0.108)	0.257 (±0.134)	0.336 (NC)	0.393 (NC)^ns^
ASP	0.50 (±0.31)	0.541 (±0.092)	0.798 (±0.057)	0.322 (±0.120)	0.411 (±0.117)^ns^
**nrITS**					
AFS	0.30 (±0.55)	0.175 (±0.069)	0.668 (±0.062)	0.737 (±0.105)	0.777 (±0.097)^ns^
West-AFS	–	–	–	–	–
East-AFS	–	–	–	–	–
AMA	0.29 (±0.37)	0.225 (±0.110)	0.997 (±0.026)	0.769 (±0.117)	0.960 (±0.032)^∗^
ACY	1.82 (±2.53)	0.182 (±0.058)	0.863 (±0.035)	0.789 (±0.066)	0.787 (±0.072)
AFR	0.83 (±0.92)	0.352 (±0.129)	0.731 (±0.096)	0.519 (±0.169)	0.506 (±0.156)
ATE	0.41 (±0.70)	0.067 (±0.042)	0.386 (±0.203)	0.827 (±0.164)	0.833 (±0.157)^ns^
ASP	1.40 (±0.85)	0.468 (±0.081)	0.538 (±0.063)	0.129 (±0.096)	0.099 (±0.085)


*Species abbreviations following Figure 1: AFS, *A. fasciculatum*; AMA, *A. mairei*; ACY, *A. cyathophorum*; AFR, *A. farreri*; ATE, *A. tetraploidum*; ASP, *A. spicatum*. –, not calculated; N_*ST*_ is significantly greater than G_*ST*_ (^∗^*P* < 0.05; ^∗∗^*P* < 0.001); ns, not significant.*

**FIGURE 2:**
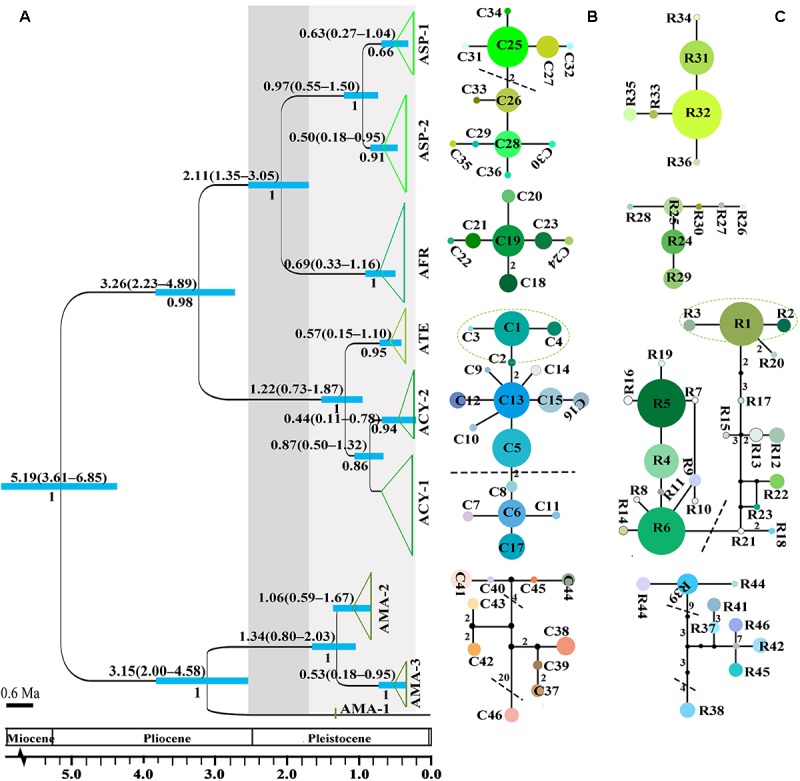
The divergence time estimation and network pattern for subgenus *Cyathophora* species based on the DNA haplotypes. **(A)** Beast-derived divergence time based on cpDNA dataset. Numbers under the branches indicate PP values and ones above the line show the mean divergence dates and 95% HPD for node ages (in Myr ago, Ma). The light blue bars indicate the 95% HPD credibility intervals. Scale bar indicating branch length of 0.6 Ma. The east–west split across 500 mm isohyet in subgenus *Cyathophora* indicates in 2.11 Ma (95% HPD, 1.35–3.05 Ma). **(B,C)** The intraspecific networks for subgenus *Cyathophora* species, respectively, based on cpDNA and nrITS haplotypes. The size of circles indicates the frequency of each haplotypes. Small solid black circles represent unsampled or extinct haplotypes. Each solid line indicates one mutational step that interconnects two haplotypes, the numbers in the solid line indicate those over one. The dotted lines indicate the intraspecific clades.

No shared chlorotypes were detected between the eastern and western genealogies both for subgenus *Cyathophora* and *A. fasciculatum* (Table 1), indicating maternal east–west break. The single-chlorotype dominant network pattern seems to be prevalent, being found in *A*. *farreri* (C19, *c*. 47.3%), *A. tetraploidum* (C1, *c*. 82.8%), the east group of *A. fasciculatum* (C56, *c*. 23.9%) and the west group of *A. fasciculatum* (C48, *c*. 69.8%) (Figure 2B, 3B). However, the pattern of clade-specific chlorotypes but no geographic break was identified in *A*. *cyathophorum* (ACY-1, C13, *c*. 22.6%; ACY-2, C6, *c*. 13.5%) and *A*. *spicatum* (ASP-1, C25, *c*. 44.9%; ASP-2, C26, *c*.17.0%), and pattern labeled by population-specific chlorotypes was detected in *A*. *mairei* (Figure 2B, 4A). C2 was found to be shared by *A. cyathophorum* (ZG24) and *A. tetraploidum* (JR4) (Figure 2B, 4A).

The cpDNA-derived AMOVA revealed a stronger population structure for each species and group (Φ_ST_ ≥ 0.59, all *P* < 0.001), except for *A. spicatum* with a weaker one (Φ_ST_ = 0.32, *P* < 0.001). For the east and west groups of *A. fasciculatum* (east/west, Φ_ST_ = 0.59/0.65, both *P* < 0.001), 25% (Φ_CT_ = 0.25, *P* < 0.001) of the total variation was partitioned to between groups, and 65% explained by variation among populations within group (Table 3).

**Table 3:** Analysis of molecular variance (AMOVA) based on cpDNA, nrITS, and nSSR datasets for each subgenus *Cyathophora* species and *A*. *fasciculatum* subdivided into west and east groups.

Species/groups	cpDNA					ITS					SSR				
			
	df.	SS	VC	PV	Φ–statistics	df.	SS	VC	PV	Φ–statistics	df.	SS	VC	PV	R–statistics
**AFS**															
Among populations	18	233.633	2.117	70.0	Φ_ST_ = 0.70^∗∗^	18	21.684	0.336	77.69	Φ_ST_ = 0.78^∗∗^	18	56767.66	249.95	59.2	R_ST_ = 0.59^∗∗^
Within populations	91	82.622	0.908	30.0		38	4	0.105	22.31		211	36340.53	172.23	40.8	
**West-AFS**															
Among populations	11	163.471	2.481	64.6	Φ_ST_ = 0.65^∗∗^	11	18.571	0.324	89.1	Φ_ST_ = 0.89^∗∗^	11	38992.11	313.91	58.8	R_ST_ = 0.59^∗∗^
Within populations	55	74.827	1.361	35.4		51	2	0.039	10.18		118	26005.02	220.38	41.2	
**East-AFS**															
Among populations	6	12.299	0.312	59.0	Φ_ST_ = 0.59^∗∗^	6	4.857	0.244	64.71	Φ_ST_ = 0.65^∗^	6	1759.72	13.26	10.7	R_ST_ = 0.11^∗^
Within populations	36	7.794	0.217	41.0		14	2	0.133	35.29		93	10335.51	111.13	89.3	
**West/East-AFS**															
Among groups	1	57.863	0.847	24.6	Φ_CT_ = 0.25^∗∗^	1	5.5	0.134	29.11	Φ_CT_ = 0.29^∗^	1	16015.83	113.54	23.9	R_CT_ = 0.24^∗^
Among populations															
Within group	17	175.77	1.685	49.0	Φ_SC_ = 0.65^∗∗^	17	23.429	0.302	51.88	Φ_SC_ = 0.83^∗∗^	17	40751.83	190.17	40.0	R_SC_ = 0.52^∗∗^
Within populations	91	82.622	0.908	26.4		65	4	0.062	19.01		211	36340.53	172.23	36.2	
**AMA**															
Among populations	6	202.954	5.961	97.6	Φ_ST_ = 0.98^∗∗^	6	251.28	7.32	93.36	Φ_ST_ = 0.93^∗∗^	6	10917.38	140.97	43.4	R_ST_ = 0.43^∗∗^
Within populations	34	4.9	0.144	2.4		34	17.7	0.521	6.64		77	14160.13	183.91	56.6	
**ACY**															
Among populations	19	162.613	0.832	75.3	Φ_ST_ = 0.75^∗∗^	14	221.91	1.362	69.01	Φ_ST_ = 0.69^∗∗^	19	36976.63	87.14	29.2	R_ST_ = 0.29^∗∗^
Within populations	180	49.157	0.273	24.7		154	94.196	0.612	30.99		380	80188.79	211.02	70.8	
**ATE**															
Among populations	5	6.305	0.129	78.6	Φ_ST_ = 0.79^∗∗^	5	4.865	0.088	39.73	Φ_ST_ = 0.39^∗∗^	5	20303.85	179.33	21.3	R_ST_ = 0.21^∗∗^
Within populations	51	1.8	0.035	21.4		51	7.1	0.139	61.27		108	71595.01	662.92	78.7	
**AFR**															
Among populations	6	27.493	0.576	76.9	Φ_ST_ = 0.77^∗∗^	6	15.485	0.336	53.47	Φ_ST_ = 0.53^∗∗^	6	6179.05	47.54	15.7	R_ST_ = 0.16^∗∗^
Within populations	48	8.289	0.173	23.1		42	12.27	0.292	46.53		109	27732.45	254.43	84.3	
**ASP**															
Among populations	11	31.37	0.26	31.7	Φ_ST_ = 0.32^∗∗^	11	12.487	0.077	13.91	Φ_ST_ = 0.14^∗∗^	11	76064.13	375.72	49.3	R_ST_ = 0.49^∗∗^
Within populations	95	53.228	0.56	68.3		92	44.09	0.479	86.09		198	76577.68	386.76	50.7	


*Species abbreviations following Figure 1: AFS, *A. fasciculatum*; AMA, *A. mairei*; ACY, *A. cyathophorum*; AFR, *A. farreri*; ATE, *A. tetraploidum*; ASP, *A. spicatum*. ^∗^*P* < 0.05; ^∗∗^*P* < 0.001, 10,000 permutations for significance.*

### The Divergence Time Estimations for Each Lineage

**FIGURE 3:**
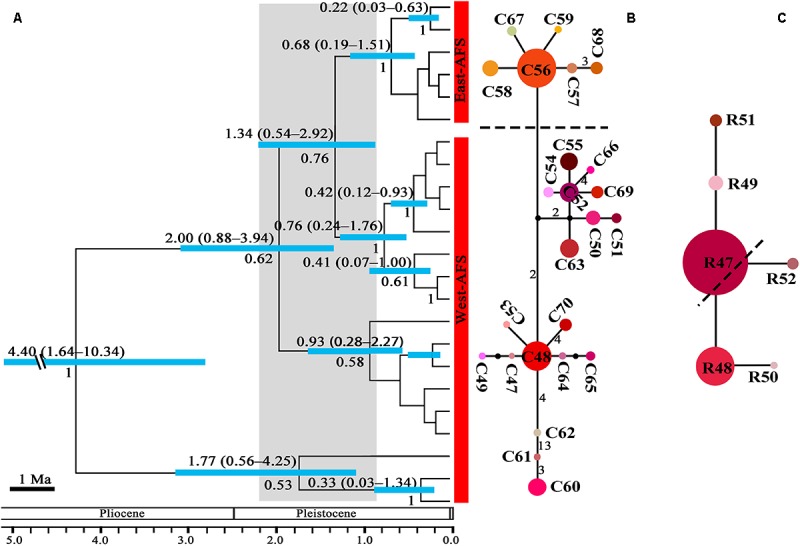
The phylogeny and divergence time estimation and network pattern for *A*. *fasciculatum* (AFS) based on DNA haplotypes. **(A)** Beast-based divergence time based on cpDNA sequences. Numbers under the branches indicate PP values and ones above the line show the mean divergence dates and 95% HPD for node ages (Ma). The light blue bars indicate the 95% HPD credibility interval. Scale bar indicating branch length of 1 Ma. The east–west split across 500 mm isohyet in *A. fasciculatum* indicates in 1.37 Ma (95% HPD, 0.54–2.92). **(B,C)** The intraspecific networks for *A. fasciculatum*, respectively, based on cpDNA and nrITS haplotypes. The size of circles indicates the frequency of each haplotypes. Small solid black circles represent unsampled or extinct haplotypes. Each solid line indicates one mutational step that interconnects two haplotypes, the numbers in the solid line indicate those over one. The dotted lines indicate the east and west clades.

The crown node of east–west split within subgenus *Cyathophora* was dated around 2.11 Ma (95% HPD, 1.35–3.05 Ma; Figure 2A), and that within *A. fasciculatum* around 1.37 Ma (95% HPD, 0.54–2.92 Ma; Figure 3A). The divergence among the clades of *A*. *mairei* was dated back to Pliocene–Pleistocene (3.15–1.34 Ma), as well as the divergence among the western clades of *A*. *fasciculatum* (4.34–1.74 Ma). The intraspecific divergence and intra-clade divergence for these species mainly were dated back to the largest Quaternary glaciation in the QTP (*c*. 1.2–0.6 Ma) (Yi et al., 2005). For example, the intraspecific divergence of *A. cyathophorum* was estimated around 1.22 Ma, that of *A. spicatum* around 0.97 Ma, that of *A. farreri* around 0.69 Ma and that of *A. tetraploidum* around 0.57 Ma (Figure 2A). For the intra-clade divergence of *A. mairei* was estimated around 1.06 to 0.53 Ma (Figure 2A), and that of the west group of *A. fasciculatum* around 0.93 to 0.76 Ma, and that of the east group of *A. fasciculatum* around 0.68 Ma (Figure 3A).

### nrITS Diversity and Network Topology

A total of 46 and 6 ribotypes based on 133 and 4 variable sites were, respectively, identified for subgenus *Cyathophora* species (Supplementary Table S9) and *A*. *fasciculatum* (Supplementary Table S10). The nrITS-based H_T_ was higher than H_S_, but lower than the cpDNA-based H_T_ (Table 2). However, no well-defined phylogeographic structures were detected in each species (all *P* > 0.05), except in *A*. *mairei* (N_ST_ > G_ST_, *P* < 0.05) (Table 2). Neutrality test statistics based on Tajima’s *D* and Fu’s Fs were generally non-significant for all taxa (Supplementary Table S8).

**FIGURE 4:**
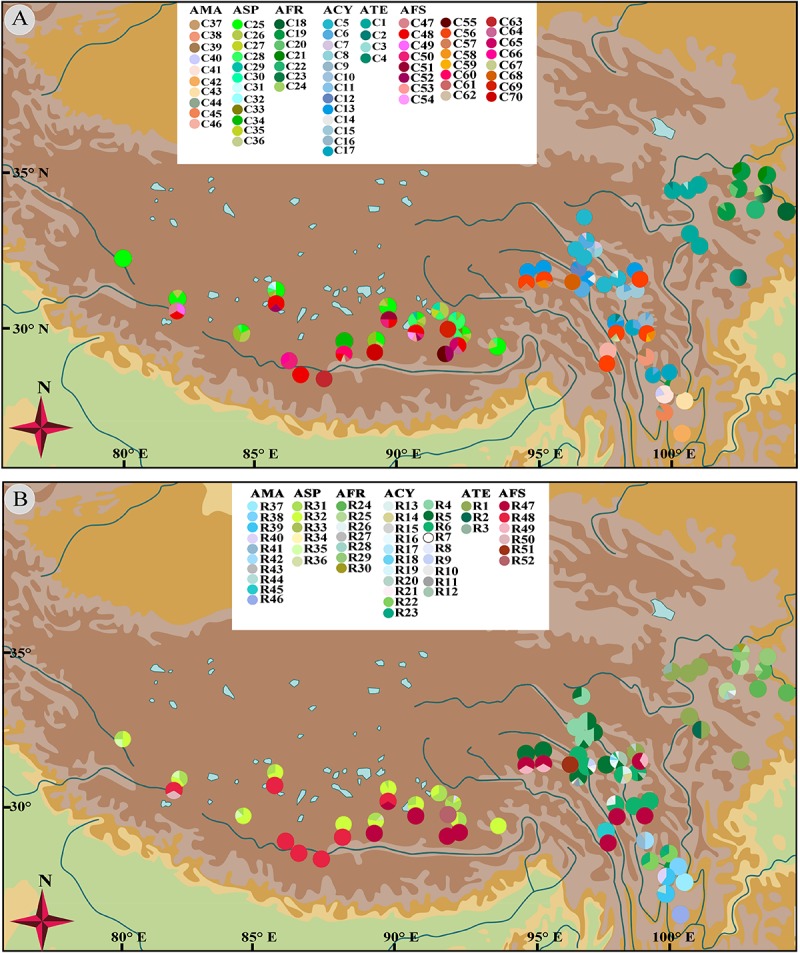
Geographical distribution of 70 chlorotypes **(A)** and 52 ribotypes **(B)** for subgenus *Cyathophora* species and *A. fasciculatum*.

No shared ribotypes were found between the eastern and western lineages of subgenus *Cyathophora*, indicating well biparental east–west break. However, the eastern dominant R47 of *A. fasciculatum* was found in the western populations, which geographically are adjacent to the 500 mm isohyet (p61, 63, 65–67) (Figure 4B), suggesting the existence of narrow genetically admix cline (Barton and Gale, 1993). The single-ribotype dominant network pattern was found approximately within each species, e.g., in *A*. *farreri* (R24, *c*. 36.7%), in *A. tetraploidum* (R1, *c*. 86.0%), in *A*. *spicatum* (R32, *c*. 51.9%), and in *A. fasciculatum* (R47, *c*. 67.3%) (Figure 2C and Table 1). Like cpDNA-based network, *A*. *cyathophorum* showed a clade-specific pattern but no geographic break (ACY-1, R5 and R6, *c*. 30.5% and 31.4%; ACY-2, no dominant ribotypes presented), and *A*. *mairei* displayed a population-specific pattern (Figure 2C, 4B). R1 was found to co-exist in one population of *A*. *cyathophorum* (GT21) and all populations of *A. tetraploidum* (Figure 4B and Table 1).

The nrITS-based AMOVA recovered a stronger population structure for each species and group (Φ_ST_ ≥ 0.53, all *P* < 0.001), except for *A. tetraploidum* (Φ_ST_ = 0.39, *P* < 0.001) and *A*. *spicatum* (Φ_ST_ = 0.14, *P* < 0.001) with a weaker such structure (Table 3). For the east and west groups of *A. fasciculatum* (east/west, Φ_ST_ = 0.65/0.89, both *P* < 0.001), 29% (Φ_CT_ = 0.29, *P* < 0.05) of the total variation was partitioned between groups, and 83% explained among populations within group (Table 3).

### Nuclear Microsatellite Diversity and Population Structure

A few null alleles were detected in subgenus *Cyathophora* and *A. fasciculatum* (Supplementary Table S11). When the null alleles were filtered, little effects on the genetic structures of subgenus *Cyathophora* and *A. fasciculatum* were showed. Thus all the nSSRs were used in the following analyses. In total, 251 and 55 alleles were, respectively, scored over nSSR loci for subgenus *Cyathophora* and *A*. *fasciculatum*. The allelic diversity was highly variable across loci, with A_O_ ranging from 1 to 15, H_O_ from 0.00 to 0.948, H_E_ from 0.00 to 0.806, H_S_ from 0.00 to 0.632, F_ST_ from –0.041 to 0.720 and F_is_ from –1.00 to 0.429 (Supplementary Table S11). The values of A_O_, H_O_, H_S_, R_S_, and F_is_ of each population across all loci ranged from 11 to 32, 0.111 to 1.00, 0.067 to 0.504, 1.3 to 2.4, and –1.00 to 0.429, respectively (Table 1). No significant genotypic linkage disequilibrium for pairwise loci was detected within any population (*P* > 0.05). The nSSR-based AMOVA demonstrated significant population structure for each species (R_ST_ ranged from 0.16 to 0.59, all *P* < 0.001). For the east and west groups of *A. fasciculatum*, 24% (Φ_CT_ = 0.24, *P* < 0.05) of the total variation was partitioned between groups, and 40% explained among populations within group (Table 3).

**FIGURE 5:**
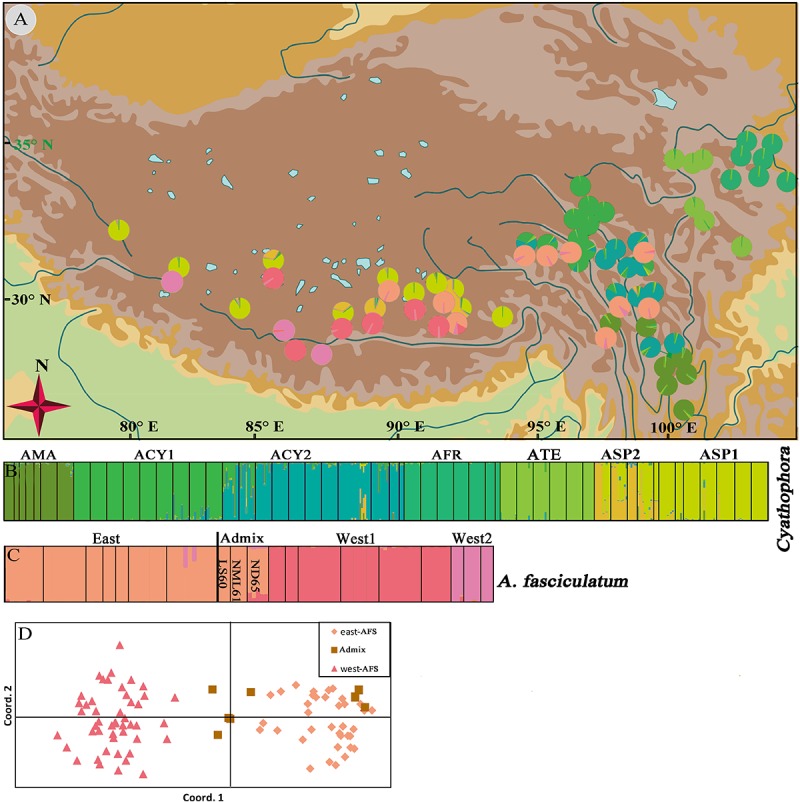
Geographic origin and color-grouping of the 52 populations of subgenus *Cyathophora* at *K* = 7, and that of 19 populations of *A*. *fasciculatum* at *K* = 3 **(A)**, and histogram of the STRUCTURE assignment test **(B,C)** and principal coordinate analysis (PCoA) for *A*. *fasciculatum* **(D)** based on nine nuclear microsatellite loci.

STRUCTURE results showed that the individuals of subgenus *Cyathophora* and *A*. *fasciculatum* can be, respectively, clustered into seven (*K* = 7) (Figure 5B) and three groups (*K* = 3) (Figure 5C) according to the highest lnP(D) and delta *K* values (Supplementary Figure S3). The geographic distribution of seven clusters of subgenus *Cyathophora* was highly congruent with that of DNA sequence (cpDNA and nrITS) in representing species (Figure 4), whereby individuals of *A*. *cyathophorum* (ACY1 and ACY2) and *A*. *spicatum* were further subdivided (ASP1 and ASP2) (Figure 5A,B). The geographic distribution of three clusters of *A*. *fasciculatum* was greatly consistent with that of cpDNA data in representing the east and west groups of *A. fasciculatum* (Figure 4A), whereby the west individuals of *A. fasciculatum* were further subdivided into West1 and West2 (Figure 5A,C). Observationally, the genetic admixture line near to the 500 mm isohyet comprising three western populations (ND65, LS60, and NML61) was also found in the STRUCTURE result (Figure 5C), as that presented in the nrITS data (Figure 4B). Likewise, this genetic admixture was also supported by the PCoA for *A*. *fasciculatum*, in despite of the absence of genetic admixture in LS60 and NML61, being thought probably due to the limited individuals for each population (Figure 5D and Table 1).

### PCAs for Ecological Differentiation, IBDs and IBEs

**FIGURE 6:**
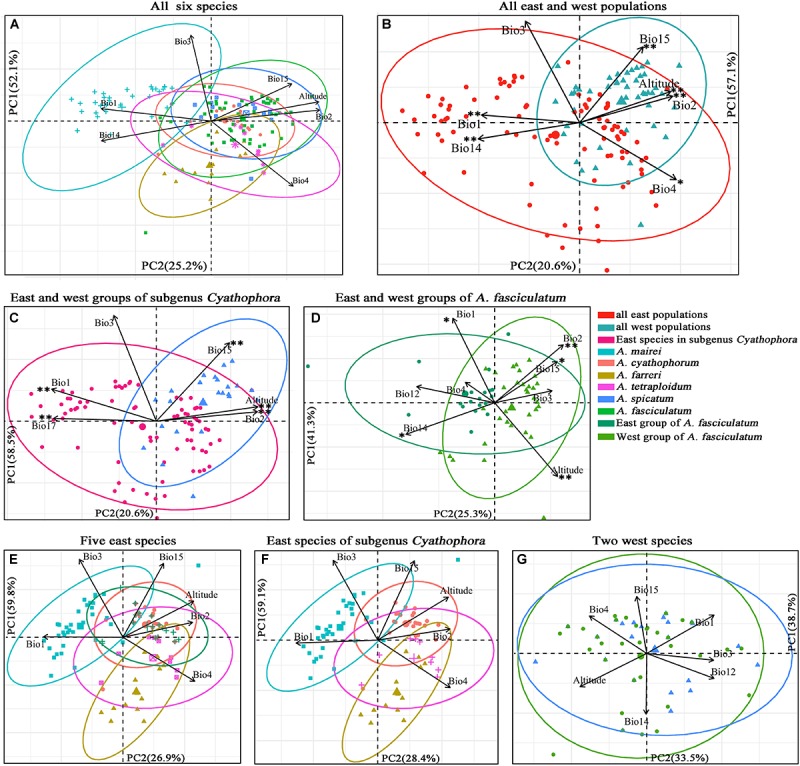
Scatter plots of PC1 and PC2 **(A–D)** showing ecological differentiation between the east and west groups, respectively, for all species, subgenus *Cyathophora* and *A. fasciculatum*, as well as **(E–G)** the niche differentiation among all east species and all west species based on the bioclimatic variables exhibiting pairwise Pearson correlation coefficients *r* < 0.7 (Supplementary Table S5). ^∗∗^*P* < 0.001, ^∗^*P* < 0.05 indicating significant difference of the bioclimatic variables between the east and west groups estimated by PERMANOVA and Tukey’s HSD tests (Supplementary Table S12).

More than ten georeferenced localities were sampled for each group of interest, with exception of *A. tetraploidum* having nine ones due to its narrow distributions (Supplementary Table S3). Results of the PCAs are exhibited in Figure 6. For all six species, the PCA recovered two components that cumulatively explained 77.3% of variation (Figure 6A). The scatter plot showed when these six species were split into east and west groups, the greater niche overlap was presented along PC1 and PC2 (Figure 6B). East group including *A. mairei*, *A. cyathophorum*, *A. farreri*, *A. tetraplodium* and the east populations of *A. fasciculatum*, occupies an ecological niche with higher annual mean temperature (Bio1), isothermality (Bio3) and precipitation of driest month (Bio14), while lower temperature seasonality (Bio4) and precipitation seasonality (Bio15), whereas west group comprising *A. spicatum* and the west populations of *A. fasciculatum*, occupies a contrast ecological niche compared to the east group. The niche overlap was also found in the east and west groups of subgenus *Cyathophora* (totally 79.1% of variation, Figure 6C) and *A. fasciculatum* (totally 66.6% of variation, Figure 6D) along PC1 and PC2. The scatter plot presented that the east group of subgenus *Cyathophora* has a distinct ecological niche with higher annual mean temperature (Bio1), isothermality (Bio3) and precipitation of driest quarter (Bio17), while the west group has an ecological niche with higher mean diurnal range (Bio2), precipitation seasonality (Bio15) and altitude (Figure 6C). For *A. fasciculatum*, the scatter plot showed that the east group has an ecological niche with higher annual mean temperature (Bio1) and precipitation of driest month (Bio14), while the west group possesses an ecological niche with higher mean diurnal range (Bio2) and altitude (Figure 6D). The large overlap of ecological niche was revealed in the sympatric species both for *A. cyathophorum* and the east group of *A. fasciculatum* (Figure 6E), as well as *A. spicatum* and the west group of *A. fasciculatum* (Figure 6G). Additionally, the geographically parapatric species in subgenus *Cyathophora* (*A. mairei* vs. *A. cyathophorum*, and *A. cyathophorum* vs. *A. farreri*) exhibited slightly overlap, while the allopatric species *A. mairei* vs. *A. farreri* showed clearly ecological niche differentiation. By contrast, the allotetraploid species *A. tetraploidum* showed great niche overlap with its potential diploid parents *A. cyathophorum* and *A. farreri*, and a larger niche breadth compared to the diploid parental progenitors (Figure 6F).

The significant difference of determinant bioclimatic variables in the specific group was approximately consistent between the AVOVA tests and Tukey’s HSD tests (Supplementary Table S12). For the east–west split among all species, Bio1, Bio2, Bio4, Bio14, Bio15 and altitude were significantly different in both tests (*P* < 0.05), while PC1 was significant difference in the Tukey’s HSD tests (*P* = 0.0137), but not in the AVOVA test (*P* > 0.05). For such east–west split in subgenus *Cyathophora*, Bio1, Bio2, Bio15, Bio17 and altitude were significantly different in both tests (*P* < 0.001). For such split within *A. fasciculatum*, Bio1, Bio12, Bio14, Bio15 and altitude were significantly different in both tests (*P* < 0.02). There were non-significant differences of the determinant bioclimatic variables in the sympatric species including the west group of *A. fasciculatum* – *A. spicatum*, and the east group of *A. fasciculatum* – *A. cyathophorum* (both *P* > 0.05) (Figure 6). The Mantel test showed no significant IBDs throughout subgenus *Cyathophora* (*r* = -0.063 *P* = 0.456), and *A. fasciculatum* (*r* = 0.057, *P* = 0.08). Likewise, no significant IBEs were detected within *A. fasciculatum* (all *P* > 0.05, Supplementary Table S13), however, the significant IBEs were identified within subgenus *Cyathophora*, whereby Bio2 (*r* = 0.132, *P* = 0.007) and Bio3 (*r* = 0.156, *P* = 0.012) could be the most relevant variables explaining the population genetic structures of subgenus *Cyathophora* (Supplementary Table S13).

### The Ecological Niche Modeling

**FIGURE 7:**
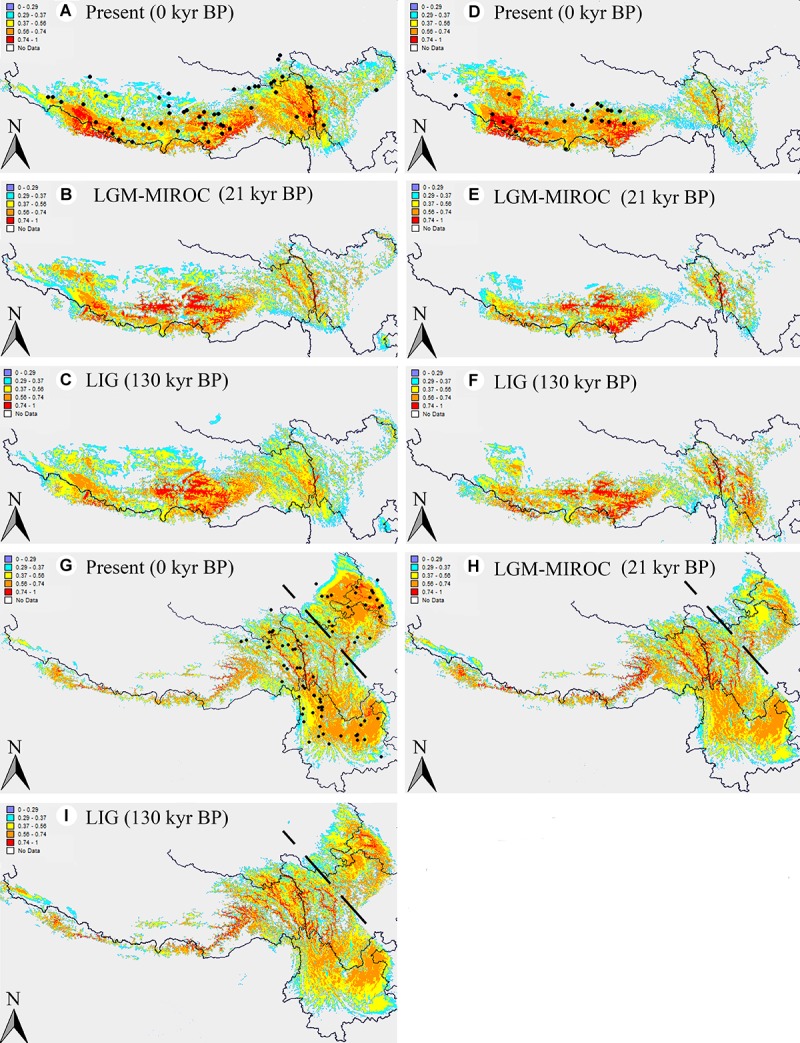
Results of ecological niche modeling of *A*. *fasciculatum* and subgenus *Cyathophora* (*A*. *spicatum*; the eastern species set including *A. mairei*, *A*. *cyathophorum*, *A*. *farreri* and *A. tetraploidum*). **(A,D,G)** Predicted distribution probability (as a logistic value) for current climatic conditions. The dark spots indicate the georeferenced localities (Supplementary Table S3). **(B,E,H)** Average projections of the model to the last glacial maximum (*c*. 21 kyr BP) using the Model for Interdisciplinary Research on Climate (MIROC) general circulation model simulations. **(C,F,I)** Average projection of the model to the last interglacial (*c*. 120–140 kyr BP). The dots signifying the species’ present georeferenced localities, and the dash line dividing the eastern species of subgenus *Cyathophora* into the northern species *A. tetraploidum* and *A*. *farreri*, and the southern species *A*. *cyathophorum* and *A*. *mairei*.

The ENM simulations based on Maxent model showed a perfect predictive power for *A*. *fasciculatum*, *A*. *spicatum*, and the eastern subgenus *Cyathophora* species, with the AUC = 0.967 ± 0.009, 0.986 ± 0.003 and 0.981 ± 0.005 (mean ± SD), respectively. The model predicted suitable habitats for these units were highly coincident with their actual geographic range (Figure 7A,D,G), except that of *A*. *spicatum* expanding to the eastern QTP, however, with a clear gap between the western and eastern distribution, which may attribute to these determinant climate variables resulting in their niche overlap (Figure 6C). Compared with the present distributions, the suitable habitats of *A*. *spicatum* and the west group of *A*. *fasciculatum* were largely contracted toward the eastern Himalayas with the lower ELAs during the LGM period, whereas this tendency was weakened during the LIG period (Figure 7A–F). The suitable habitats of the east group of *A*. *fasciculatum*, *A*. *farreri*, and *A. tetraploidum*, respectively, were largely contracted *in situ* during the LGM, however, no significant changes for that of *A*. *mairei* and *A*. *cyathophorum* (Figure 7A,B,G,H).

## Discussion

### Climatic-Induced Phytogeographic Divide Along the 500 mm Isohyet in the QTP

The morphology clearly demonstrates that there are two distinctive groups for the genus *Allium* subgenus *Cyathophora*, which well correspond to the eastern monsoonal and the western continental climatic zones in the QTP across the 500 mm isohyet. Of which, the eastern group with umbel inflorescence comprises *A*. *mairei*, *A*. *cyathophorum*, *A*. *farreri* and *A. tetraploidum*, while the western group with spicate inflorescence only includes *A*. *spicatum* (Figure 1). However, *A*. *fasciculatum* which co-exists with *A. cyathophorum* in the eastern QTP, and with *A. spicatum* in the western QTP, shows no obvious morphological variation (Supplementary Figure S1). Strikingly, the genetic breaks both in subgenus *Cyathophora* and in *A*. *fasciculatum* strongly supported this climatically east–west split across the 500 mm isohyet (Figure 4, 5). Nonetheless, this split in the nuclear genealogy of *A*. *fasciculatum* has been obscured most likely due to the genetic introgression indicated by the narrow genetically admix cline (Barton and Gale, 1993), which geographically was adjoining to the 500 mm isohyet (Figure 4B, 5A). The significant phylogeographical structures in cpDNA data but not in nrITS data for most species strongly supported the limited seed flows but frequent pollen flows among these *Allium* populations in the QTP (Table 2). In combination with the expansion of the derived R47 (Figure 3C) from the east to the west within *A. fasciculatum*, it is suggested the pollen flows from the east group to the west group in *A. fasciculatum* could have resulted in this narrow genetic admixture line (Figure 3), which reasonably explains why the genetic admixture only was detected in the nuclear genealogy but not in the cpDNA genealogy (Rieseberg and Soltis, 1991).

The divergence time estimations for this east–west break were traced back to the early Pleistocene (*c*. 2.11 Ma for subgenus *Cyathophora*; *c*. 1.37 Ma for *A*. *fasciculatum*) (Figure 2, 3), which predates the largest glacial age in the QTP (*c*. 1.2–0.6 Ma), but coincidently falls into the climatic condition differentiation in the QTP around 3.6–1.2 Ma (Liu and Dong, 2013), suggesting the east–west genetic breaks within subgenus *Cyathophora* and *A. fasciculatum* could have been caused by the climatic differences. This hypothesis is also supported by the distinct climatic niches between the east and west groups (Figure 6B) determined by the significantly different bioclimatic variables (Supplementary Table S12) both in subgenus *Cyathophora* and *A. fasciculatum* (Figure 6). Noticeably, the larger niche overlap between the east and west groups (Figure 6), probably suggests the shallow or intermediate climatic-derived divergence (Antonelli, 2017). However, no significant correlations between the genetic divergence and bioclimatic variables were found within *A. fasciculatum*, but the significantly ones within subgenus *Cyathophora* (Supplementary Table S13). Considering the striking morphological differentiations between the east (umbel inflorescence, larger seeds) and west (spicate inflorescence, smaller seeds) groups of subgenus *Cyathophora* (Figure 1) (Li et al., 2016), it is assumed this climatic-induced genetic beak could have been deepened in subgenus *Cyathophora* due to the prezygotic sterility resulted from the morphological vicariance (e.g., Landmann and Winding, 1993; Kennedy et al., 2012; Kou et al., 2014; Wen et al., 2014). However, no such case occurs between the east and west groups of *A. fasciculatum* (Supplementary Figure S1), it is asserted that the frequent pollen flows within *A. fasciculatum* could have obscured this climatic-induced genetic break (Figure 4B, 5A).

To sum up, it seems reasonable to support that the climatic-derived 500 mm isohyet acts as a natural line of vicariant event to partition subgenus *Cyathophora* and *A*. *fasciculatum* into western and eastern genealogies during the climatic difference episode.

### Divergence Caused by Tectonic Uplift and Quaternary Climatic Changes

Our phylogenetic analyses indicated that *A. fasciculatum* could have ancestrally located in the western QTP (Figure 3), and subgenus *Cyathophora* ancestrally settled in the eastern QTP (Figure 2) (Li et al., 2016). In terms of ‘center–periphery’ hypothesis, it is predicted a higher genetic diversity, lower genetic differentiation and more constant population size in the ancestral areas, but not in the recolonized areas (Eckert et al., 2008). The DNA sequence-based AMOVA recovered a higher genetic differentiation (Φ_ST_) and total genetic diversity (H_T_) in the ancestral areas than those in the recolonized areas both for subgenus *Cyathophora* (eastern Φ_ST_ ≥ 0.77, western Φ_ST_ = 0.32 for cpDNA; eastern Φ_ST_ ≥ 0.39, western Φ_ST_ = 0.14 for nrITS) and *A. fasciculatum* (western Φ_ST_ = 0.65, eastern Φ_ST_ = 0.59 for cpDNA; western Φ_ST_ = 0.89, eastern Φ_ST_ = 0.65 for nrITS) (Tables 2, 3). Based on the cpDNA sequences, a higher average genetic diversity within populations (H_S_ = 0.479) and a larger effect population size inferred from the nucleotide diversity (π = 0.93 × 10^-3^) were found in the western ancestral group of *A. fasciculatum*, when comparing to the eastern derived group (H_S_ = 0.287 and π = 0.14 × 10^-3^) (Table 2). Conversely, it was revealed that all the eastern species of subgenus *Cyathophora* have lower average genetic diversity within populations (H_S_ ≤ 0.381) and smaller effect population size (π ≤ 0.24 × 10^-3^) when comparing to the western species *A. spicatum* (H_S_ = 0.541 and π = 0.50 × 10^-3^) (Table 2). The BEAST-based divergence estimations showed that the crown divergence at the ancestral areas both for subgenus *Cyathophora* (5.19–3.26 Ma; Figure 2A) and *A*. *fasciculatum* (4.34–1.74 Ma; Figure 3A) was estimated to occur around the Pliocene–Pleistocene, coinciding to the tectonic motions of the QTP (An et al., 2014). It is thus inferred that the tectonic uplift could have facilitated greatly for the genetic differentiation among populations in the ancestral areas, and the ancestral effect population of subgenus *Cyathophora* could have been subdivided into different small parts. This tectonic uplift-driven diversification has been increasingly recognized as an important process that can have diverse outcomes with respect to the biodiversity in the QTP (e.g., Favre et al., 2015; Zhang et al., 2016; Xing and Ree, 2017).

The nSSRs-based AMOVA revealed a lower genetic differentiation both for subgenus *Cyathophora* (eastern R_ST_ ≤ 0.43, western R_ST_ = 0.49) and *A. fasciculatum* (western R_ST_ ≤ 0.59, eastern R_ST_ = 0.11). Compared to cpDNA and nrITS, microsatellite markers usually have faster mutation rates and thus are more related to more recent population history. The intraspecific divergence within subgenus *Cyathophora* (1.06–0.69 Ma; Figure 2A) and the intra-clade divergence of *A. fasciculatum* (0.69–0.33 Ma; Figure 3A) were mainly dated back to the largest Quaternary glaciation in the QTP (*c*. 1.2–0.6 Ma), probably highlighting the importance of Quaternary glaciations to their recent diversification in the QTP (Liu et al., 2014; Wen et al., 2014; Du et al., 2017).

Of the east species, the ENMs showed larger contractions of the suitable habitats, respectively, for *A. farreri*, *A. tetraploidum*, and *A. fasciculatum* during the LGM (Figure 7), suggesting a contraction–expansion pattern for these species when they responded the Quaternary glacial oscillations. The single dominant haplotype among populations, respectively, within *A. farreri* (C19/R24), *A. tetraploidum* (C1/R1) and *A. fasciculatum* (C56/R47) (Figure 2, 3) most probably indicates the species specific glacial refugium, and the population diversifications could have been resulted in by this contraction–expansion pattern. For *A. mairei*, no clear contraction of suitable habitat during the LGM (Figure 7), the greatly dominant population-specific haplotypes (Figure 2) and significantly phylogeographical structure (Table 2), suggest that it could have survived from the dispersed mountain glacial refugia resulted from tectonic uplift in its early divergence (Figure 2A). For *A*. *cyathophorum*, two genetic lineages (Figure 2, 5B) possibly indicate two isolated glacial refugia (Figure 2). However, the striking lack of phylogeographic structure within *A*. *cyathophorum* (G_ST_ > N_ST_, *P* > 0.05) (Table 2), possibly suggests that the frequent gene flows could have blurred the imprints of refugial isolation. However, this is challenged by the range displacement most likely due to the invasion of *A. tetraploidum* to *A. cyathophorum* (the genetic information from *A. tetraploidum* was detected in p9 of *A. cyathophorum*, Li et al., 2016). The STRUCTURE-based result showed that the p7–11 of the ACY1, were uniquely sub-clustered, but not for the p12–16 of ACY1 and p17–21 of ACY2, which contained genetic information from both lineages, presumably indicating the formation of a moving hybrid zone due to the genetic admixture from ACY1 to ACY2 (Figure 5A) (Godbout et al., 2012; Cinget et al., 2015). The wider niche for *A. tetraploidum* than the parental progenitors (Figure 6F), meaning the stronger ability to inhabit different conditions, and to use different resources (Carrillo-Angeles et al., 2016), probably provides a prerequisite for this invasion. In contrast, a significantly phylogeographical structure was recovered in the east population of *A. fasciculatum* (Table 2), nevertheless the sympatric distribution and similar niches to *A. cyathophorum* (Figure 6E). Considering their approximately same divergence time (Figure 2A, 3A), it is suggested that the range displacement could have a deep influence on the genetic structure of *A. cyathophorum*.

Of the west species, the ENMs presented the same contraction–expansion pattern, respectively, for the suitable habitats of *A. spicatum* and *A. fasciculatum* when responding the glacial crisis (Figure 7), which could have contributed much to the divergence of *A. spicatum* (Figure 2A) and the intra-clade divergence of *A. fasciculatum* (Figure 3A). However, the phylogeographic structures (Table 2) and AMOVA results indicate a distinct population genetic structures between *A. fasciculatum* and *A. spicatum* (Table 3), notwithstanding their similar niches (Figure 6G). It deems this pattern can be much attributed to the key role of the tectonic motions during the Pliocene–Pleistocene in the early divergence of *A*. *fasciculatum* (Figure 3A). This proposal is also supported by the similar genetic structures between *A. mairei* and the west group of *A. fasciculatum* (Table 3), most likely due to the tectonic motions for their early inter-clade divergence (Figure 2A, 3A). The contracted ranges of *A*. *spicatum* and *A. fasciculatum* during the LGM indicate their refugia would locate at eastern Himalayas with lower ELAs (Figure 7). This strongly supports the local survival of *A*. *spicatum* and *A. fasciculatum* from the Quaternary freezing damages in the western QTP, agreeing with some tolerant cold organisms, such as *Aconitum gymnandrum* (Wang et al., 2009), *Juniperus tibetica* complex (Opgenoorth et al., 2010), *Hippophae thibetana* (Wang et al., 2010), *Orinus thoroldii* (Liu et al., 2015) and *Eospalax baileyi* (Tang et al., 2010). It is implied that the aridification in the western QTP possibly could have reduced the prevalence of large ice sheets, characterized by the higher ELAs (Figure 1) (Shi et al., 1998; Shi, 2002), which in turn provides a promise for the local survival from the glacial crisis.

Taken together, it is inferred that the eastern species have the species specific glacial refugia and consequently resulted in the diversifications *in situ*, most probably due to the development of the dispersed mountain glaciations. By contrast, the western species mostly could have largely contracted to the eastern Himalayas where the larger ice sheet could have limited due to the drought in the western QTP. In this way, the distinct responses to the Quaternary climatic oscillations could have been recovered for species restricted to different climatic zones. Additionally, the early divergence trigged by the tectonic motions and genetic introgression could have a deep effect on the species’ genetic structures.

## Conclusion

Our chloroplast and nuclear DNA results for subgenus *Cyathophora* and *A*. *fasciculatum*, along with the present environmental space, robustly support the existence of a biogeographic divide between the eastern and western QTP along the 500 mm isohyet. The causative factor for this genetic divide is suggested to be the climatic differentiation in the eastern and western QTP. Interestingly, this climate-mediated split within subgenus *Cyathophora* could have been deepened due to the prezygotic sterility resulted from the morphological vicariance from the east umbel inflorescence to the west spicate inflorescence, while that within *A*. *fasciculatum* could have been obscured due to the pollen flows from the east to west caused by the postglacial expansion. It is recovered that the tectonic motions could be responsible for the ancestral divergence, and the Quaternary climatic fluctuations for the recent divergence. Strikingly, distinct responses to the Quaternary climatic fluctuations have been revealed in the components of the eastern and western QTP, which could be closely related with the climatic differences. Overall, our findings highlight the importance of orogeny and climatic changes in the QTP in shaping species diversification during the Pliocene–Pleistocene.

## Author Contributions

ML, DX, CX, and YD conducted fieldwork. ML and XH conceived the research. ML and YZ performed the experiments. ML, DX, YY, and YD analyzed the data. ML wrote the manuscript. All authors read and reviewed the manuscript.

## Conflict of Interest Statement

The authors declare that the research was conducted in the absence of any commercial or financial relationships that could be construed as a potential conflict of interest.

## Footnotes

1 http://www.bio-soft.net/tree/Network.htm

2 http://tree.bio.ed.ac.uk/software/figtree

3 http://www.worldclim.org/current
