# Supplementary material for: A Phytogeographic Divide Along the 500 mm Isohyet in the Qinghai-Tibet Plateau: Insights From the Phylogeographic Evidence of Chinese Alliums (Amaryllidaceae)
Source: Front Plant Sci. 2019 Mar 5;10:149. doi: 10.3389/fpls.2019.00149 (PMC6412145; doi:10.3389/fpls.2019.00149)
Supplement: Supplementary file 1 [file Data_Sheet_1.pdf]

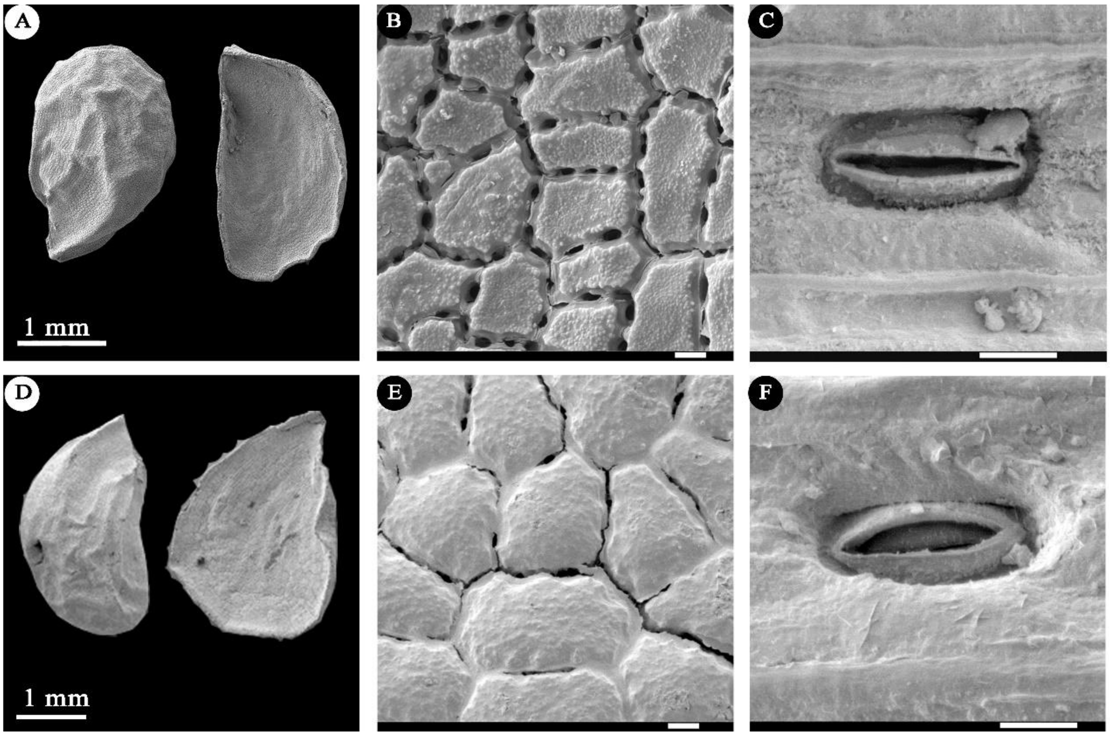

Figure S1 Scanning electron micrographs of seeds and stomata of the east (A–C) and west (D–F) groups of *A. fasciculatum*.

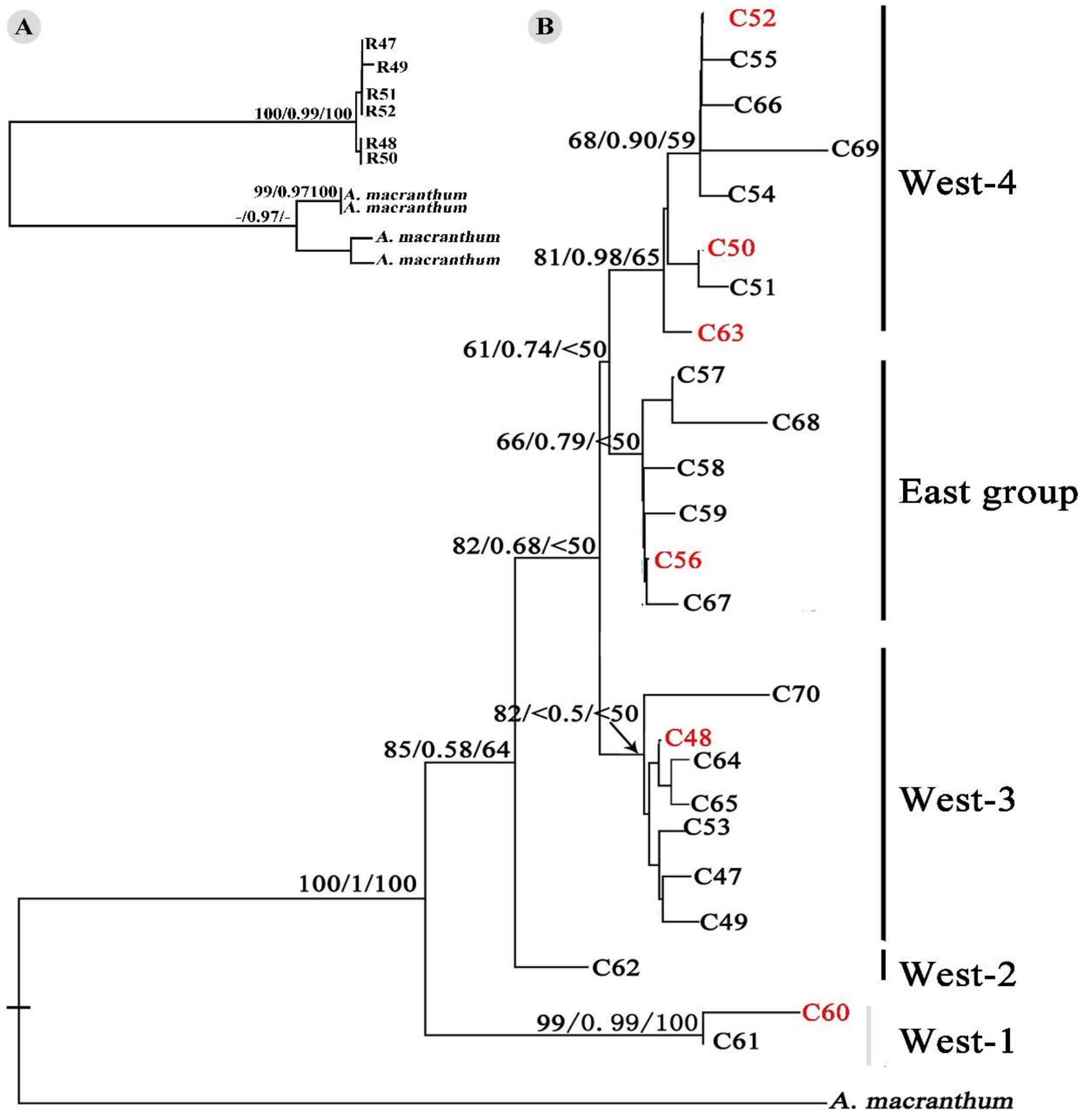

Figure S2 The monophyly of *A. fasciculatum* recovered from the haplotypes of nrITS and cpDNA with *A. macranthum* as outgroups. The Bayesian inference (BI) tree is only shown for the similar topologies among maximum parsimony (MP) tree and Neighbor-joining (NJ) tree. Numbers on the branches indicate NJ/PP/BP, respectively.

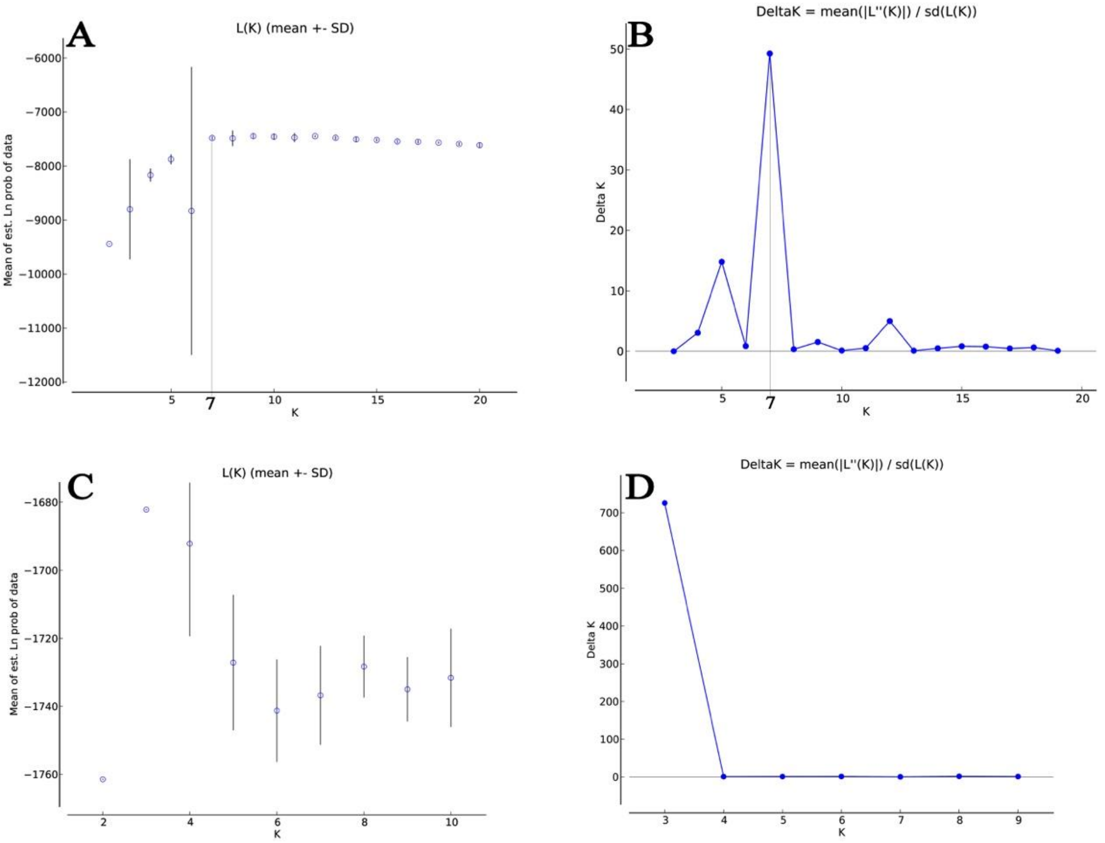

Figure S3 The dot plot represents changes of the mean posterior probability (loge P(D) (±SD) values of each K according to Pritchard et al. (2000) (A, C) and the line diagram indicates the corresponding DK statistics according to Evanno et al. (2005) (B, D).

Table S1 The detailed sample localities and sample of cpDNA and nrITS in each population in this study. The abbreviation for each species and population corresponding to those in the main test. Species abbreviation in the whole paper: *A. mairei* AMA; *A. cyathophorum* ACY; *A. farreri* AFR; *A. tetraploidum* ATE; *A. spicatum* ASP; *A. fasciculatum* AFS.

Species	Acronym	Sample location	Voucher specimen	Geographical coordinate	Alt. (m)	Sample number	cpDNA samples	nrITS samples
**ATE**	LH1	Xiaojin xian,Lianghe, SC	LMJ2013070244	N31.4849; E102.4922	3650	7	7	7
	BM2	Banma xian, QH	LMJ2013071205	N32.9327; E100.7371	3611	10	10	10
	MKR3	Banma xian, Mark river original forest, QH	LMJ2013071307	N32.6542; E100.9757	3300	10	10	10
	JR4	Dawu xian, jiangrang hydropower,QH	LMJ2013071510	N34.7127; E100.2422	3450	10	10	10
	LJ5	Maqin xian, lajiazhen, QH	LMJ2013071614	N34.6795; E100.6405	3090	10	10	10
	HB6	Tongde xian, hebei xiang saiqinagou,QH	LMJ2013071716	N34.7619; E100.8930	3329	10	10	10
**ACY**	XSM7	Yushu xian,Xiaosumang,QH	LMJ2013072536	N33.7670; E96.7446	4183	10	10	10
	JXF8	Yushu xian,jiangxi forest station,QH	LMJ2013072537	N32.3082; E96.8887	3583	10	10	10
	MZ9	Nangqian xian,maozhuang xiang,QH	LMJ2013072538	N32.2666; E96.8246	4200	10	10	10
	MN10	Maozhuang xiang to Nangqian xian,QH	LMJ2013072539	N32.2318; E96.6406	4200	10	10	10
	KD11	Maozhuang xiang to kanda cun, QH	LMJ2013072540	N32.2166; E96.8246	4057	10	10	10
	LX12	Leiwuqi, apart from xilang cun 40 Km, Tibet	LMJ2013072641	N31.5209; E96.5990	3700	10	10	10
	LW13	Leiuiqi xian, Tibet	LMJ2013072642	N31.2116; E96.6002	3737	10	10	10(12)
	LK14	Leiwuqi xian,kamaduo xiang, Tibet	LMJ2013072743	N31.1561; E96.4744	3750	10	10	10(23)
	DQ15	Dingqing xian, shangyi cun, Tibet	LMJ2013072844	N31.6723; E95.0582	3700	10	10	10
	RB.16	Suoxian, Rongbuzhen, Tibet	LMJ2013072845	N31.5677; E94.6726	3797	10	10	10
	XP17	Gongjue xian, xiangpi xiang, Tibet	LMJ2013080252	N31.0145; E98.2417	3811	10	10	8(9)
	GJ18	Gongjue xian, Tibet	LMJ2013080253	N31.0299; E98.4045	3704	10	10	10
	TB19	Changdu , tuoba xiang, dari cun, Tibet	LMJ2013080354	N31.2822; E97.5262	3900	10	10	10
	JY20	Jiangda , jueyong cun, Tibet	LMJ2013080355	N31.3394; E97.9272	3837	10	10	9(16)
	GT21	jiangda , gangtuozhen, Tibet	LMJ2013080356	N31.6272; E98.5856	3826	10	10	10

	ZB22	Mangkang, zhubalong, Tibet	LMJ2013082879	N29.7249; E98.7416	3412	10	10	10
	MK23	Mangkang, Tibet	LMJ2013082880	N29.7248; E98.5316	3805	20	20	20
	ZG24	Zuogong, wangdajie military depot, Tibet	LMJ2013082984	N29.6730; E97.8475	3805	11	10	10
	WF25	Zhongdian xian, Wufeng Mt.,YN	y11100401	N27.8046; E99.7169	3350	6	7	7
	YZ26	Zhongdian xian, yizhong,YN	y11100406	N27.8266; E99.6933	3267	3	3	3
**AFR**	ZQ27	Zhouqu, heiyugou,GS	LMJ2013081369	N33.9181; E104.1839	2300	10	10	7
	DB28	Diebu, wangzang forest,GS	ZCJ2011072103	N34.0705; E103.2383	2537	3	10	1
	KL29	Kangle xian, lianhuashan, GS	ly20130701	N35.0909; E103.6873	3290	10	10	7
	ZN30	Zhuoni xian, GS	ly20130702	N34.6177; E103.5426	3650	10	1	10
	XH31	Xiahe xian, qingshui,GS	T2013080808	N35.3066; E102.6252	2800	6	10	5(6)
	LQ32	Luqu, langmuqiao,GS	LMJ2013081472	N34.9405; E102.5669	2800	10	10	9
	MQ33	Maqu, waixiangsi,GS	LMJ2013081575	N33.9469; E102.0387	3148	10	6	9
**ASP**	LS34	Lhasa, Kitayama, Tibet	LMJ2013090390	N29.9774; E91.6798	3596	10	10	9(12)
	ND35	Naidong xian, party school, Tibet	LMJ2013100998	N29.1823; E91.7692	3900	10	10	10(16)
	LZX36	Linzhou xian, Tibet	xz2011082701	N30.2668; E91.2405	4000	5	5	3
	LZ37	Lazi xian, Tibet	LMJ2013090496	N29.1264; E87.6291	3726	3	1	1
	NM38	Nanmulin xian,Tibet	LMJ2013090792	N30.3699; E89.3385	4045	13	13	13
	RK39	Rikeze xian,Tibet	xz2011082609	N29.2198; E88.8363	4588	6	6	6(9)
	CQ40	Cuoqin xian,racetrack,Tibet	LMJ2013090593	N31.0631; E85.1629	3900	10	10	10(16)
	DJ41	DianJiao, GaEr, Tibet	lmj2014100226	N32.6261; E79.5205	4385	10	10	10
	HE42	HuoEr, PuLan, Tibet	lmj2014100327	N30.9863; E81.6147	4532	10	10	10
	ZB43	Xin ZhongBa, Tibet	lmj2014100429	N29.7341; E84.0390	4544	11	11	11
	LKZ44	Zangquweiyuanhui, LangKaZi, Tibet	lmj2014100834	N29.9612; E90.3403	4469	12	12	12
	ML45	RixuCun, Milin, Tibet	lmj2014101441	N29.1268; E93.4467	3052	10	10	10
**AMA**	CY46	ChaYu xian, Tibet	XZ2011081611	N28.7934; E97.4978	2715	5	5	5
	BS47	Baishaxiang, Lijiangshi, YN	y11092623	N27.0294; E100.21	2906	3	3	3

	WB48	Weibazhicun, Habaxueshan, Xianggelalashi, YN	y11100602	N27.3684; E100.1447	2852	4	4	4
	LJS49	Laojunshan, Jianchuanshi, YN	y11100802	N26.6228; E99.6096	3324	5	5	5
	CS50	Cangshan, Dalishi, YN	y11101003	N25.6831; E100.1268	2864	4	4	4
	ZD51	Yicu xianrendong, Zhongdianxian, YN	LY2013081101	N27.8263; E99.7230	3100	10	10	10
	DQ52	Baimaxueshan, Deqinxian, YN	LY2013081202	N28.4977; E98.9397	4231	10	10	10
**AFS**	JD53	jiangda , gangtuozhen, Tibet	LMJ2013080358	N31.6272; E98.5856	3826	4	4	4
	MK54	Mangkang, zhubalong, Tibet	LMJ2013082883	N29.7245; E98.7416	3441	9	9	9
	ZG55	Zuogong，wangdajie military depot, Tibet	DYQ20140825	N29.6730; E97.8475	3800	10	10	10
	DQ56	Dingqing xian, Sezhaxiang, Tibet	LMJ2013072847	N31.6723; E95.0582	3700	10	10	10
	RB57	Suoxian, Rongbuzhen, Tibet	LMJ2013072846	N31.5677; E94.6726	3797	4	4	4
	CY58	Sangjiucun, ChaYuxian, Tibet	XZ2011081912	N28.6231; E97.4922	3300	3	3	3
	LWQ59	Changmaoling, Leiwuqi, Tibet	10–1328	N31.2903; E96.4597	3903	3	3	3
	LS60	Lhasa, Kitayama, Tibet	xz2011082705	N29.9774; E91.6798	3596	3	3	3
	NML61	layacun, jiacuoxiang, NanMuLin, Tibet	lmj2014100632	N30.3699; E89.3385	4142	4	4	4
	CQ62	CuoQin,Tibet	LMJ2013090594	N31.0631; E85.1629	4588	4	4	4
	RKZ63	Rikeze xian,Tibet	xz2011082608	N29.2198; E88.8363	4588	3	3	3
	LZ64	Lazi xian, Tibet	LMJ2013090795	N29.1264; E87.6291	4000	10	10	10
	ND65	Naidong xian, party school, Tibet	LMJ2013090999	N29.1823; E91.7692	4000	5	5	5
	LKZ66	Zangquweiyuanhui, LangKaZi, Tibet	lmj2014100833	N29.9612; E90.3403	4497	10	10	10
	CM67	Cuomei, Tibet	lmj2014101136	N28.8944; E91.6258	4131	11	11	11
	JN68	The fork of Jilong and NieLaMu	DYQ2014090802	N28.5605; E85.6024	3726	3	3	3
	NLM69	NieLaMu, Tibet	LMJ2013090897	N28.1605; E85.9732	3865	7	7	7
	ZF70	North mountainas of Qomolangma	DYQ2014090901	N27.9800; E86.9230	3900	4	4	4
	HE71	HuoEr, PuLan, Tibet	lmj2014100328	N30.9863; E81.6147	4500	3	3	3

SC, Sichuan Province; QH, Qinghai Province; XZ, Xizang Autonomous Region; GS, Gansu Province; YN, Yunnan Province. The number following Acronym is the population natural location corresponding to Figure 4–1. The number in parentheses is clones for nrITS in sequence number column.

Table S2 The primers of three cpDNA fragments and nrITS and 9 pairs microsatellite markers, and their amplified protocols. For each primer pair, repeat motif and size of cloned allele (bp) were listed.

Gene name	Repeat Motif<br>Allele range (bp)	Primers(5'–3')	Protocols
rps16	/	rps16F–AAA CGA TGT GGT ARA AAG CAA C	94 °C for 4min; 30 cycles × 94 °C for 45 s, 52 °C for 45 s, and 72 °C for 1min; and 72 °C for 10 min
	/	rps16R–AAC ATC WAT TGC AAS GAT TCG ATA	
trnD–trnT	/	trnD^{GUC}F–ACC AAT TGA ACT ACA ATC CC	94 °C for 4min; 30 cycles × 94 °C for 45 s, 60 °C for 1 min, and 72 °C for 1min; and 72 °C for 10 min
	/	trnT^{GGU}R–CTA CCA CTG AGT TAA AAG GG	
trnL–trnF	/	trnL^{UAA}F–CGA AAT CGG TAG ACG CTA CG	94 °C for 4min; 30 cycles × 94 °C for 45 s, 60 °C for 1 min, and 72 °C for 1min; and 72 °C for 10 min
	/	trnF^{GAA} R–ATT TGA ACT GGT GAC ACG AG	
ITS	/	ITS4–TCC TCC GCT TAT TGA TAT GC	94 °C for 3min; 30 cycles × 94 °C for 45 s, 55 °C for 45 s, and 72 °C for 1min; 72 °C for 10 min;
	/	ITS5–GGA AGT AAA AGT CGT AAC AAG G	
GB–AS–001	(TA)4<br>180–308	F–TCTTTAGGGCTGAGTTGGC——FAM	94 °C for 2min; 30 cycles × (94 °C for 45 s, 57 °C for 30 s, and 72 °C for 45 s); 10 cycles × (94 °C for 45 s, 54 °C for 30 s, and 72 °C for 45 s); and 72 °C for 10 min
		R–TCCTTCTTAGTTGGCGGC	
GB–AS–011	(CCT)4e(CT)3<br>267–327	F–CAATTGAAGATCGTCGGC——HEX	94 °C for 2min; 30 cycles × (94 °C for 45 s, 58(–48) °C for 30 s, and 72 °C for 45 s); 10 cycles × (94 °C for 45 s, 55(–45) °C for 30 s, and 72 °C for 45 s); and 72 °C for 10 min
		R–CCGATTTGGGTGTAGGGT	
GB–AS–046	(GTG)3<br>147–168	F–GGGTGCACGAAACTACCA——ROX	94 °C for 2min; 30 cycles × (94 °C for 45 s, 54 °C for 45 s, and 72 °C for 30 s); 10 cycles × (94 °C for 45 s, 51 °C for 45 s, and 72 °C for 30 s); and 72 °C for 10 min
		R–TCGTTAAATCGAACCTAACCC	
GB–AS–063	(CGG)5<br>230–278	F–AGAGAGCTTCCTTGCGGT——FAM	94 °C for 2min; 30 cycles × (94 °C for 45 s, 59 °C for 45 s, and 72 °C for 45 s); 10 cycles × (94 °C for 45 s, 56 °C for 45 s, and 72 °C for 45 s); and 72 °C for 10 min
		R–CCTCCAAACTTCCGACCT	

GB–AS–089	(AG)4e, (TAG)3<br>343–409	F–GTCCAAGGAGGCAAGGAC——FAM<br>R–GGCTAGCCAATCCATTCC	94 °C for 2min; 30 cycles × (94 °C for 45 s, 56 °C for 45 s, and 72 °C for 1min); 10 cycles × (94 °C for 45 s, 53 °C for 45 s, and 72 °C for 1min); and 72 °C for 10 min
GB–AS–102	(AAAT)3<br>171–219	F–AATCATCTTCGGGCCACT——ROX<br>R–CCTAGAACGAGTGTGAAGGG	94 °C for 2min; 30 cycles × (94 °C for 45 s, 56 °C for 45 s, and 72 °C for 30 s); 10 cycles × (94 °C for 45 s, 53 °C for 45 s, and 72 °C for 30 s); and 72 °C for 10 min
GB–AS–109	(ACC)4<br>190–208	F–GGTCTCCTCATCCACCGT——ROX<br>R–GTGTGGGGCATGATTGAC	94 °C for 2min; 30 cycles × (94 °C for 45 s, 53 °C for 45 s, and 72 °C for 30 s); 10 cycles × (94 °C for 45 s, 51 °C for 45 s, and 72 °C for 30 s); and 72 °C for 10 min
GB–AS–112	(TA)5<br>218–256	F–GTTGCAGGTCCAGGTTCA——HEX<br>R–ACCAGCAACAACACAGGG	94 °C for 2min; 30 cycles × (94 °C for 45 s, 57 °C for 45 s, and 72 °C for 30 s); 10 cycles × (94 °C for 45 s, 54 °C for 45 s, and 72 °C for 30 s); and 72 °C for 10 min
eSSR6	(CCT)2C4T3C3<br>190–222	F–GATTCCTGGTTGAAGTCAGAGG——FAM<br>R–CTCACTTCTCTTTCGCCTCAAT	94 °C for 2min; 30 cycles × (94 °C for 45 s, 59 °C for 45 s, and 72 °C for 45 s); 10 cycles × (94 °C for 45 s, 56 °C for 45 s, and 72 °C for 30 s); and 72 °C for 10 min

Table S3 T The detailed information of the georeferenced localities used to execute the bioclimatic variations for PCA and ENMs.

Species	Latitude	Longitude	Species	Latitude	Longitude	Species	Latitude	Longitude
A. tetraploidum	N31.4849	E102.4922	*A. spicatum*	N29.2198	E88.8363	*A. fasciculatum*	N31.6272	E98.5856
A. tetraploidum	N32.9327	E100.7371	*A. spicatum*	N31.0631	E85.1629	*A. fasciculatum*	N29.7245	E98.7416
A. tetraploidum	N32.6542	E100.9757	*A. spicatum*	N32.6261	E79.5205	*A. fasciculatum*	N29.6730	E97.8475
A. tetraploidum	N34.7127	E100.2422	*A. spicatum*	N30.9863	E81.6147	*A. fasciculatum*	N31.6723	E95.0582
A. tetraploidum	N34.6795	E100.6405	*A. spicatum*	N29.7341	E84.0390	*A. fasciculatum*	N31.5677	E94.6726
A. tetraploidum	N34.7619	E100.8930	*A. spicatum*	N29.9612	E90.3403	*A. fasciculatum*	N29.9774	E91.6798
A. tetraploidum	N31.7948	E103.5415	*A. spicatum*	N29.1268	E93.4467	*A. fasciculatum*	N30.3699	E89.3385
A. tetraploidum	N35.5536	E99.8450	*A. spicatum*	N29.9693	E91.9500	*A. fasciculatum*	N31.0631	E85.1629
A. tetraploidum	N32.22040	E101.27798	*A. spicatum*	N30.4397	E91.0306	*A. fasciculatum*	N29.2198	E88.8363
A. cyathophorum	N33.7670	E96.7446	*A. spicatum*	N29.2369	E90.6311	*A. fasciculatum*	N29.1264	E87.6291
A. cyathophorum	N32.3082	E96.8887	*A. spicatum*	N29.8983	E92.5102	*A. fasciculatum*	N29.1823	E91.7692
A. cyathophorum	N32.2666	E96.8246	*A. spicatum*	N29.0781	E92.7886	*A. fasciculatum*	N29.9612	E90.3403
A. cyathophorum	N32.2318	E96.6406	*A. spicatum*	N29.0749	E92.7883	*A. fasciculatum*	N28.8944	E91.6258
A. cyathophorum	N32.2166	E96.8246	*A. spicatum*	N29.2619	E91.3153	*A. fasciculatum*	N28.5605	E85.6024
A. cyathophorum	N31.5209	E96.5990	*A. spicatum*	N28.5605	E85.6024	*A. fasciculatum*	N28.1605	E85.9732
A. cyathophorum	N31.2116	E96.6002	*A. spicatum*	N28.8640	E85.2977	*A. fasciculatum*	N27.9800	E86.9230
A. cyathophorum	N31.1561	E96.4744	*A. spicatum*	N29.1963	E84.7585	*A. fasciculatum*	N30.9863	E81.6147
A. cyathophorum	N31.6723	E95.0582	*A. spicatum*	N29.4347	E90.1628	*A. fasciculatum*	N28.6231	E97.4922
A. cyathophorum	N31.5677	E94.6726	*A. spicatum*	N27.3841	E88.9174	*A. fasciculatum*	N31.2903	E96.4597
A. cyathophorum	N31.0145	E98.2417	*A. mairei*	N28.7934	E97.4978	*A. fasciculatum*	N29.1047	E99.6425
A. cyathophorum	N31.0299	E98.4045	*A. mairei*	N27.0294	E100.21	*A. fasciculatum*	N31.4803	E93.6801
A. cyathophorum	N31.2822	E97.5262	*A. mairei*	N27.3684	E100.1447	*A. fasciculatum*	N31.1976	E89.3191
A. cyathophorum	N31.3394	E97.9272	*A. mairei*	N26.6228	E99.6096	*A. fasciculatum*	N30.9777	E81.2880
A. cyathophorum	N31.6272	E98.5856	*A. mairei*	N25.6831	E100.1268	*A. fasciculatum*	N29.8708	E92.6221

A. cyathophorum	N29.7249	E98.7416	*A. mairei*	N27.8263	E99.7230	*A. fasciculatum*	N30.9331	E88.7076
A. cyathophorum	N29.7248	E98.5316	*A. mairei*	N28.4977	E98.9397	*A. fasciculatum*	N29.6721	E91.3483
A. cyathophorum	N29.6730	E97.8475	*A. mairei*	N26.0862	E103.1858	*A. fasciculatum*	N28.9014	E88.0201
A. cyathophorum	N27.8046	E99.7169	*A. mairei*	N25.1021	E102.6953	*A. fasciculatum*	N28.5360	E92.8847
A. cyathophorum	N27.8266	E99.6933	*A. mairei*	N25.0685	E102.6244	*A. fasciculatum*	N29.1277	E92.8171
A. cyathophorum	N29.1047	E99.6425	*A. mairei*	N25.1200	E102.7025	*A. fasciculatum*	N28.9044	E85.3854
A. cyathophorum	N31.9264	E99.1997	*A. mairei*	N24.7817	E103.1924	*A. fasciculatum*	N29.8275	E92.3447
A. cyathophorum	N29.8940	E97.6327	*A. mairei*	N25.0501	E102.5898	*A. fasciculatum*	N30.2708	E94.8161
A. cyathophorum	N28.3370	E99.0777	*A. mairei*	N24.7309	E102.6927	*A. fasciculatum*	N32.3070	E84.0604
A. cyathophorum	N30.0016	E101.9550	*A. mairei*	N26.5420	E98.9156	*A. fasciculatum*	N29.0519	E92.9753
A. farreri	N33.9181	E104.1839	*A. mairei*	N26.9390	E98.9837	*A. fasciculatum*	N29.3988	E85.0748
A. farreri	N34.0705	E103.2383	*A. mairei*	N27.0318	E100.2624	*A. fasciculatum*	N28.7146	E89.9401
A. farreri	N35.0909	E103.6873	*A. mairei*	N27.5913	E100.1176	*A. fasciculatum*	N30.4813	E83.6537
A. farreri	N34.6177	E103.5426	*A. mairei*	N26.3570	E100.2004	*A. fasciculatum*	N30.0952	E90.5436
A. farreri	N35.3066	E102.6252	*A. mairei*	N27.7311	E98.6664	*A. fasciculatum*	N31.4388	E103.1648
A. farreri	N34.9405	E102.5669	*A. mairei*	N24.6192	E99.3558	*A. fasciculatum*	N31.6778	E95.3043
A. farreri	N33.9469	E102.0387	*A. mairei*	N27.0001	E100.1981	*A. fasciculatum*	N27.9946	E91.9562
A. farreri	N34.4395	E104.0343	*A. mairei*	N26.8248	E100.2351	*A. fasciculatum*	N31.6428	E98.7951
A. farreri	N34.0487	E104.3907	*A. mairei*	N25.1755	E100.3510	*A. fasciculatum*	N32.3082	E96.8887
A. farreri	N33.2633	E104.2332	*A. mairei*	N24.4868	E100.7318	*A. fasciculatum*	N30.6585	E82.1501
A. farreri	N35.7973	E104.0619	*A. mairei*	N25.0114	E100.0021	*A. fasciculatum*	N29.4882	E86.2683
A. farreri	N34.9414	E103.7639	*A. mairei*	N26.0101	E99.0099	*A. fasciculatum*	N29.9127	E89.3890
A. farreri	N31.8517	E102.6814	*A. mairei*	N27.0061	E100.0024	*A. fasciculatum*	N33.7670	E96.7446
A. spicatum	N29.9774	E91.6798	*A. mairei*	N27.0005	E100.0035	*A. fasciculatum*	N28.8687	E93.3785
A. spicatum	N29.1823	E91.7692	*A. mairei*	N23.6084	E104.3386	*A. fasciculatum*	N32.0514	E96.3567
A. spicatum	N30.2668	E91.2405	*A. mairei*	N25.0359	E101.5444	*A. fasciculatum*	N33.4835	E96.6231

A. spicatum	N29.1264	E87.6291	*A. mairei*	N26.2231	E104.1021	*A. fasciculatum*	N31.8977	E96.1272
A. spicatum	N30.3699	E89.3385	*A. mairei*	N25.7738	E102.9255	*A. fasciculatum*	N30.0079	E99.1094

Table S4 Bioclimatic variables.

Variable	Description	Variable	Description
BIO1	Annual mean temperature	BIO11	Mean temperature of coldest quarter
BIO2	Mean diurnal range (mean of monthly (max temp – min temp))	BIO12	Annual precipitation
BIO3	Isothermality (BIO2/BIO7) (× 100)	BIO13	Precipitation of wettest month
BIO4	Temperature seasonality (SD × 100)	BIO14	Precipitation of driest month
BIO5	Max temperature of warmest month	BIO15	Precipitation seasonality (coefficient of variation)
BIO6	Min temperature of coldest month	BIO16	Precipitation of wettest quarter
BIO7	Temperature Annual Range (BIO5–BIO6)	BIO17	Precipitation of driest quarter
BIO8	Mean temperature of wettest quarter	BIO18	Precipitation of warmest quarter
BIO9	Mean temperature of driest quarter	BIO19	Precipitation of coldest quarter
BIO10	Mean temperature of warmest quarter		

Table S5 The final used bioclimatic variables after selecting using the pairwise Pearson correlation coefficients r < 0.7 for 20 bioclimatic variables within five species of subgenus *Cyathophora* and *Allium fasciculatum*.

Species/group	Bioclimatic variable (Bio)									
A. fasciculatum	1	2	3	4	12	-	14	15	-	altitude
East group of *A. fasciculatum*	1	2	3	4	12		14	15	-	altitude
West group of *A. fasciculatum*	1	-	3	4	12	-	14	15	-	altitude
A. spicatum	1	-	3	4	12	-	14	15	-	altitude
A. cyathophorum vs. *A. tetraploidum*	1	2	3	4	-	13	-	15	-	altitude
A. farreri vs. *A. tetraploidum*	1	2	-	4	12	-	-	15	-	altitude
East species of subgenus *Cyathophora*	1	2	3	4	-	-	-	15	-	altitude
Subgenus *Cyathophora* and *A. fasciculatum*	1	2	3	4	-	-	14	15	-	altitude
Subgenus *Cyathophora*	1	2	3	-	-	-	-	15	17	altitude
East species	1	2	3	4	-	-	-	15	-	altitude
East group of *A. fasciculatum* vs. *A. cyathophorum*	1	2	3	4	12	-	14	15	-	altitude
West species	1	-	3	4	12	-	14	15	-	altitude

Table S6 Variable sites of the combined sequences of three cpDNA fragments (rps16, trnL–trnF and trnD–trnT) of subgenus *Cyathophora* species identifying forty–six chlorotypes.

Species	Variable sites								
Chlorotype	10	20	30	40	50	60	70	80	90
C1	CGATAAATTA	AATTGCTCCT	GGTTAGAGTT	TTAAACCCCC	CCCTACTAGA	CAAGATTTAA	AGTCTTAATA	ACCTTTGTCG	AAAAAGATTTA
C2	....	....	....	....	....	....	........G.	....	....
C3	....	....	....	....	..A.......	....	....	....	....
C4	....	.T........	....	....	....	....	........G.	....	..T....G...
C5	....	.T........	....	....	....GT....	....	........G.	....	..T........
C6	....	TT........	....	....	....GT....	....	........G.	........T.	..T........
C7	....	.T........	....	....	....GT....	....	........G.	....	..T....G...
C8	....	.T........	....	A.........	....	....	........G.	....	..T........
C9	....	TT........	....	....	....	....	........G.	........T.	..T........
C10	....	.T........	....	A.........	....GT....	....	........G.	....	..T........
C11	....	.T........	....	....	....	....	........G.	....	C.T........
C12	....	.T........	....	....	....	....	........G.	....	..T........
C13	....	.T........	....	....	....	....	........G.	....	..T.......G
C14	....	.T........	....	....	.A........	....	........G.	....	.TT........
C15	....	.T........	....	....	....	....	........G.	....	.TT........
C16	....	.T........	....	....	....GT....	....	........G.	....	..T.......G
C17	....	..A.......	....	....	....	....	....	....	....
C18	.......A..	G.A...C...	.T........	A...C.....	....GT....	.......G..	......T.GC	....A..C..	TTT.CA.....
C19	.......A.T	G.A...C...	.T.A......	A...C.....	....GT....	.......G..	......T.GC	....A..C..	TTT.CA.....
C20	..TA...A.T	G.A...C...	.T.A......	A...C.....	....GT....	.......G..	......T.GC	....A..C..	TTT.CA.....
C21	...A...A.T	G.A...C...	.T.A......	A...C.....	....GT....	.......G..	......T.GC	....A..C..	TTT.CA.....
C22	...A...A.T	G.AA..C...	.T.A......	A...C.....	....GT....	.......G..	......TTGC	....A..C..	TTT.CA.....
C23	.......A.T	G.A...C...	.T.A......	A...C.....	....GT....	.......G..	......T.GC	....A..C..	TTT.CA...A.
C24	...A...A.T	G.A...C...	.T.A......	A...C.....	....GT....	.......G..	......T.GC	....A..C..	TTT.CA...A.
C25	.......A.T	G.AAA.CT..	.TAA......	A........G	T...GT....	.......G..	.....A..G.	....A..C..	T..........

C26	.......A.T	G.AAA.CT..	.TAA......	A........G	T.........	.......G..	.....A..G.	....A..C..	T..........
C27	.......A.T	G.AAA.CT..	.TAA......	A........G	T...GT....	.......G..	.....A..G.	....A..C..	T.T........
C28	.......A.T	G.AAA.CT..	.TAA......	A........G	T.........	.......G..	.....A..G.	....A..C..	T.T........
C29	.......A.T	G.AAA.CT..	.TAA......	A........G	T......T..	.......G..	.....A..G.	....A..C..	T.T........
C30	.......AAT	G.AAA.CT..	.TAA......	A........G	T.........	.......G..	.....A..G.	....A..C..	T.T........
C31	.......AAT	G.AAA.CT..	.TAA......	A........G	T...GT....	.......G..	.....A..G.	....A..C..	T..........
C32	.......AAT	G.AAA.CT..	.TAA......	A........G	T...GT....	.......G..	.....A..G.	....A..C..	T.T........
C33	.......A.T	G.AAA.CT..	.TAA......	A...C....G	T.........	.......G..	.....A..G.	....A..C..	T..........
C34	.......A.T	G.AAA.CT..	.TAA......	A........G	T...GT.T..	.......G..	.....A..G.	....A..C..	T..........
C35	.......A.T	G.AAA.CT..	.TAA......	A........G	T.....AT..	.......G..	.....A..G.	....A..C..	T.T........
C36	.......A.T	G.AAA.CT..	.TAA......	A........G	T.....A...	.......G..	.....A..G.	....A..C..	T.T........
C37	TT.....A.T	G.A..AC.A.	TT..TATA.A	AGCT.T.T..	...GGT..TC	TC..TA..T.	..A...T.G.	T.GAAGA..T	......G....
C38	TT.....A.T	G.A..AC.A.	TT..TATA.A	AGCT.T.T..	...G....TC	TC.CTA..T.	..A...T.G.	..GAAGA..T	......G....
C39	TT.....A.T	G.A..AC.A.	TT..TATA.A	AGCT.T.T..	...G....TC	TC..TA..T.	..A...T.G.	T.GAAGA..T	......G....
C40	TT..TT.A.T	G.A..AC...	TT..TATA.A	AGCT.T.T..	...G....TC	TC..TAG.T.	..A.A.T.G.	..GAAGA..T	...G..G....
C41	TT..TT.A.T	G.AA.AC...	TT..TATAAA	AGCT.T.T..	...G....TC	TC..TAG.T.	..A.A.T.G.	..GAAGA..T	...G..G....
C42	TT.....A.T	G.A..AC...	TT..TATA.A	AGCT.T.T..	...G....TC	TCC.TAC.T.	..A...T.G.	..GAAGA..T	..T...G....
C43	TT.....A.T	G.A..AC...	TT..TATA.A	AGCT.T.T..	...G....TC	TCC.TA..T.	.AC...T.G.	..GAAGA..T	......G....
C44	TT..TT.A.T	G.AA.AC...	TT..TATAAA	AGCT.T.T..	...G....TC	TC..TA..T.	..ATA.T.G.	..GAAGA..T	...G..G....
C45	TT..TT.A.T	G.AA.AC...	TT..TATAAA	AGCT.T.T..	...G....TC	TC..TA..T.	..A.A.T.G.	..GAAGA..T	...G..G....
C46	.T....CA.T	GGAA.AC..G	.T....T.AA	AG...TT.T.	...G....TC	TC..TA..TT	G.A...T.G.	.TGAAGA..C	........G..

The variable sites (1–91) in the combined cpDNA sequences (2249 bp): 15, 39, 83, 88, 96, 97, 104, 114, 144, 164, 165, 166, 167, 175, 210, 213, 240, 246, 258, 271, 284, 300, 324, 386, 387, 498, 513, 571, 577, 578, 579, 583, 591, 617, 629, 677, 680, 685, 728, 754, 807, 811, 812, 849, 866, 867, 889, 895, 907, 908, 914, 935, 954, 996, 1005, 1031, 1037, 1058, 1078, 1081, 1291, 1410, 1446, 1487, 1503, 1533, 1534, 1535, 1549, 1553, 1578, 1594, 1608, 1680, 1734, 1756, 1771, 1781, 1786, 1833, 1863, 1876, 1877, 1901, 1961, 2062, 2065, 2076, 2115, 2164, 2193.

Table S7 Variable sites of the combined sequences of three cpDNA fragments (rps16, trnL–trnF and trnD–trnT) of *A. fasciculatum* identifying twenty-four chlorotypes.

Chlorotype	Variable sites																																								
	24	40	66	67	77	84	147	216	227	267	286	289	426	504	517	580	641	678	680	681	700	725	745	749	774	831	839	840	906	936	1008	1179	1287	1417	1502	1518	1625	1678	1684	1701	1711	2157
C47	T	G	T	A	T	T	T	A	G	A	A	G	A	T	T	C	C	T	T	G	C	T	T	A	C	T	T	T	T	C	T	C	C	T	T	T	C	T	C	C	T	G
C48	.	.	.	.	.	.	.	.	.	.	.	.	.	.	.	.	.	.	.	.	.	.	.	.	.	.	.	.	.	.	.	.	.	.	C	.	.	.	.	.	.	.
C49	.	.	.	.	.	.	.	.	.	.	.	.	.	G	.	.	.	.	.	.	.	.	.	.	A	.	.	.	.	.	.	.	.	.	.	.	.	.	.	.	.	.
C50	.	.	.	.	.	.	.	T	.	.	T	.	.	.	.	.	.	.	.	.	.	.	.	.	A	.	.	.	.	A	.	G	.	.	C	.	.	.	.	.	.	.
C51	.	.	.	.	.	.	.	T	.	.	T	.	.	G	.	.	.	.	.	.	.	.	.	.	A	.	.	.	.	A	.	G	.	.	C	.	.	.	.	.	.	.
C52	.	.	.	.	.	.	.	T	A	.	T	.	.	.	.	.	.	.	.	.	.	.	.	.	A	.	.	.	.	A	.	.	.	.	C	.	.	.	.	.	.	.
C53	.	.	.	.	.	.	.	.	.	.	.	.	.	G	.	.	.	.	.	.	.	.	.	.	.	.	.	.	.	.	.	.	.	.	C	.	.	.	.	.	.	.
C54	.	.	.	.	.	.	.	T	A	.	T	.	.	.	.	.	T	.	.	.	.	.	.	.	A	.	.	.	.	A	.	.	.	.	C	.	.	.	.	.	.	.
C55	.	.	.	.	.	.	.	T	A	.	T	.	.	.	.	.	.	.	.	.	.	.	.	.	A	.	.	.	.	A	.	.	.	.	C	.	T	.	.	.	.	.
C56	.	.	.	.	.	.	.	T	.	.	.	.	.	.	.	T	.	.	.	.	.	.	.	.	A	.	.	.	.	.	.	.	.	.	C	.	.	.	.	.	.	.
C57	.	.	.	.	.	.	.	T	.	.	.	.	.	.	.	T	.	.	.	.	.	.	.	.	A	.	G	.	.	.	.	.	.	.	C	.	.	.	.	.	.	.
C58	A	.	.	.	.	.	.	T	.	.	.	.	.	.	.	T	.	.	.	.	.	.	.	.	A	.	.	.	.	.	.	.	.	.	C	.	.	.	.	.	.	.
C59	.	A	.	.	.	.	.	T	.	.	.	.	.	.	.	T	.	.	.	.	.	.	.	.	A	.	.	.	.	.	.	.	.	.	C	.	.	.	.	.	.	.
C60	.	.	.	.	.	.	.	T	.	T	T	A	T	.	G	.	.	A	A	A	T	C	.	.	A	.	.	.	C	.	C	.	T	C	C	A	.	.	.	T	C	T
C61	.	.	.	.	.	.	.	T	.	T	T	A	T	.	G	.	.	A	A	A	T	C	.	.	A	.	.	.	C	.	C	.	T	C	C	A	.	.	.	.	.	.
C62	.	.	.	.	.	.	.	.	.	.	.	.	.	.	.	.	.	.	.	.	.	.	.	.	.	.	.	.	.	.	C	.	T	C	C	A	.	.	.	.	.	.
C63	.	.	.	.	.	.	.	T	.	.	T	.	.	.	.	.	.	.	.	.	.	.	.	.	A	.	G	.	.	A	.	.	.	.	C	.	.	.	.	.	.	.
C64	.	.	.	.	.	.	.	T	.	.	.	.	T	.	.	.	.	.	.	.	.	.	.	.	A	.	.	.	.	.	.	.	.	.	C	.	.	.	A	.	.	.
C65	.	.	.	.	.	.	.	.	.	.	.	.	.	.	.	.	.	.	.	.	.	.	.	.	.	.	.	.	.	.	.	.	.	.	C	.	.	.	A	.	.	.
C66	.	.	.	.	.	.	.	T	A	.	T	.	.	.	.	.	.	.	.	.	.	.	.	T	A	.	.	.	.	A	.	.	.	.	C	.	.	.	.	.	.	.
C67	.	.	.	.	.	.	.	T	.	.	.	.	.	.	.	T	T	.	.	.	.	.	.	.	A	.	.	.	.	.	.	.	.	.	C	.	.	.	.	.	.	.
C68	.	.	.	.	.	.	.	T	.	.	.	.	.	.	.	T	.	.	.	.	.	.	A	.	A	A	G	G	.	.	.	.	.	.	C	.	.	.	.	.	.	.
C69	.	.	.	T	.	A	G	T	A	.	T	.	.	.	.	.	.	.	.	.	.	.	.	.	A	.	.	.	.	A	.	.	.	.	C	.	.	G	.	.	.	.

C70	A	A	A	.	A	.	.	.	.	.	.	.	.	.	.	.	.	.	.	.	.	.	.	.	.	.	.	.	.	.	.	.	.	.	C	.	.	.	.	.	.	.

Table S8 The neutrality tests for subgenus *Cyathophora* species and *A. fasciculatum* based on cpDNA.

DNA genes	cpDNA				nrITS			
taxon	Fu's Fs	*P*	Tajima'sD	*P*	Fu's Fs	*P*	Tajima'sD	*P*
A. fasciculatum	0.603	N.A.	0.037	0.753	0.098	N.A.	0.000	1.000
West group of *A. fasciculatum*	0.973	N.A.	0.172	0.796	0.105	N.A.	0.000	1.000
East group of *A. fasciculatum*	-0.392	N.A.	-0.415	0.479	0.100	N.A.	0.000	1.000
A. mairei	0.182	N.A.	-0.273	0.727	0.634	N.A.	0.376	0.925
A. cyathophorum	0.384	N.A.	-0.14	0.728	0.547	N.A.	0.067	0.732
A. tetraploidum	-0.113	N.A.	-0.371	0.72	0.464	N.A.	0.381	0.975
A. farreri	0.373	N.A.	0.288	0.807	-0.247	N.A.	0.140	0.767
A. spicatum	-0.269	N.A.	0.226	0.673	0.883	N.A.	0.647	0.337

Table S9 Variable sites of the nrITS for subgenus *Cyathophora* species identifying forty–six ribotypes.

Ribotype	Variable sites				
	1 12 24	34 45 57	64 75 87	94 105 117	124
	* * *	* * *	* * *	* * *	*
R1	TCACATTCTTACATCACGAGGATGACATGATTA	TAGATTAATCATAGTTACTTGGGAAGGAAC	TCACTGCGACGTAAGTACGAACGTATCATT	CGTCGGCTGACGTCTACGTCCTTGTTCCTT	TTGTGTATGG
R2	....	....C.........................	.........................C....	....	....
R3	....	.........................A....	....	....	....
R4	....................T...T.T...A..	...T.......T.....A..C.........	..........AGG.....A...........	............G..T..............	....
R5	....................T...T.T...A..	...T.......T.....A..C.........	...G......AGG.....A...........	............G..T..............	....
R6	........................T.T......	...T.......T.....A..C.........	..........AGG.....A...........	............G..T..............	....
R7	........................T.T...A..	...T.......T.....A..C.........	...G......AGG.....A...........	............G..T..............	....
R8	..C.....................T.T......	...T.......T.....A..C.........	..........AGG.....A...........	............G..T..............	....
R9	........................T.T......	...T.......T.....A..C.........	...G......AGG.....A...........	............G..T..............	....
R10	........................T.T......	...T......G.T.....A..C........	...G......AGG.....A...........	............G..T..............	....
R11	....................T...T.T......	...T.......T.....A..C.........	..........AGG.....A...........	............G..T..............	....
R12	.......................AT.T......	...T...T...T.....A............	..........A.......A...........	............A..T............C.	....
R13	........................T.T......	...T...T...T.....A............	..........A.......A...........	............A..T............C.	....
R14	........................T.T......	...T.......T.....A..C.........	..........AGG.....A...........	.......A....G..T..............	....
R15	..C.....................T.T.C....	...T.......T.....A............	..........A.......A...........	.......A....A..T..............	....
R16	....................T...T.T...A..	..A........T.....A..C.........	...G......AGG.....A...........	............G..T..............	....
R17	..........................T......	.................A............	..........A.......A...........	............A.................	....
R18	.......................AT.T......	...T...T...T.....A............	..........AGG.....A...........	............G..T..............	....
R19	....................T...T.T...A..	...T.......T.....A..C.........	...G......AGG.....A...........	............A..T............C.	....
R20	....	..A...........................	....	....	....
R21	........................T.T......	...T.......T.....A............	..........AGG.....A...........	............G..T..............	....
R22	........................T.T......	...T.......T.....AC...........	..........A.......A...........	.....T......G..T..............	....
R23	........................T.T......	...T.......T.....AC...........	..........AG......A...........	............G..T..............	....
R24	..........G.......A....T.T......	........CA..T..CTT.........G..	.....AG.G.A...C...........T.C.	..........A......T............	....
R25	..........G.......A....T.T......	........CA..T..CTT.........G.T	.....AG.G.A...C...........T.C.	..........A......T............	....

R26	..........G........A....T.T......	........CA..T..CTT........CG.T	.....AG.G.A....C..........T.C.	....C......AA......T..........	....
R27	..........G........A....T.T......	........CA..T..CTT........CG.T	.....AG.G.A....C..........T.C.	...........AA......T..........	....
R28	..........G........A....T.T......	........CA..T..CTT.........G.T	.....AG.G.A....C..........T.C.	....C......A.......T..........	....
R29	..........G........A....T.T......	........CA..T..CTT.........G..	.....AG.GTA....C..........T.C.	...........A.......T..........	....
R30	..........G........A....T.T......	........CA..T..CTT.........G.T	.....AG.G.A....C..........T.C.	...........AA......T..........	....
R31	C....CC.C..T...GT.GACC..TTT......	......C.CAT.T...T.A........GG.	AT........A.T.....ATG.T...TGA.	.ACT..TC...A..AT.C...A..C.....	..A.AG....
R32	C.......C..T...GT.GACC..TTT......	......C.CAT.T...T.A........GG.	AT........A.T.....ATG.T...TGA.	.ACT..TC...A..AT.C...A..C.....	..A.AG....
R33	C.......C..T...GT.GACCA.TTT......	......C.CAT.T...T.A........GG.	AT........A.T.....ATG.T...TGA.	.ACT..TC...A..AT.C...A..C.....	..A.AG....
R34	C....CC.C..T...GT.GACC..TTT......	......C.CAT.T...T.A........GG.	AT........A.T.....ATGTT...TGA.	.ACT..TC...A..AT.C...A..C.....	..A.AG....
R35	C.......C..T...GT.GACCA.TTT......	......C.CAT.T...T.A........GG.	AT........A.T.....ATGTT...TGA.	.ACT..TC...A..AT.C...A..C.....	..A.AG....
R36	C.......C..T...GT.GACC..TTT......	......C.CAT.T...T.A........GG.	AT........A.T.....ATGTT...TGA.	.ACT..TC...A..AT.C...A..C.....	..A.AG....
R37	AT.TT..T.G...GTGTTGACC..TTT..G.CG	AG.T.A.....GTAC.T.A.T..GC..GG.	.T..CA.A..A..GA.TGTGTGT...T.AC	T.........A...C.T...GG.TCCTAAC	.C.G..CC.A
R38	AT..T..T.....GTCT.GACC..TTT..G.A.	AG...A.....GTA..T.G.T..GC..GG.	.TG.C..A..A..GA.TGAGTAT...T.AC	T.....T...A...C.T...GG.TCCTAAC	C.....T...
R39	AT..C..T....CGTGT.GACC..TTTC.G.C.	AG...A.....GTA..T.A.T..GC..GC.	.T..C..A..A..GA.TGAGTAT.G.T.GC	T.........A...C.....GG.TCCTAAC	.C.G..C..A
R40	AT..C..T....CGTGT.GACC..TTTC.G.C.	AG...A.....GTA..T.A.T..GC..GC.	.T..C..A..A..GA.TGAGTAT.G.T.GC	T.........A...C...C.GG.TCCTAAC	.C.G..C..A
R41	AT.TT..T.G...GTGTTGACC..TTT..G.CG	AG.T.A.....GTAC.T.A.T..GC..GG.	.T..CA.A..A..GA.TGTGTGTG..T.AC	T........GA...C.T...GG.TCCTAAC	.C.G..CCTA
R42	AT.TT..T.G...GTGT.GACC..TTT..G.CG	AG.T.A.....GTAC.T.A.T..GC..GG.	.T..CA.A..A..GA.TGCGTGT...T.AC	T.........A...C.T...GGCTCCTAAC	.C.G..CC.A
R43	AT.TT..T.G...GTGT.GACC..TTT..G.CG	AG.T.A.....GTAC.T.A.T..GC..GG.	.T..CA.A..A..GA.TGCGTGT...T.AC	T.........A...C.T...GG.TCCTAAC	.C.G..CC.A
R44	AT..C..T....CGTGT.GACC..TTTC.G.C.	AG...A.....GTA..T.A.T..GC..GC.	.T..C..A..A..GA.TGAGTAT.G.T.GC	T.........A...C.....GG.TCCTAAC	.C.G..CC.A
R45	AT.TT..T.G...GTGT.GACC..TAT..G.CG	AG.T.A.....GTAC.T.A.T..GC..GG.	.T..CA.A..A..GA.TGCGTGT...T.AC	T.........A...C.T...GG.TCCTAAC	.C.G..CC.A
R46	AT.TT..T.....G.GT.GACC..TTT..G.CG	AG.T.A.....GTAC.T.A.T.CGC..GG.	.T..CA.A..A..GA.TGCGTGT...T.AC	T.........AA..C.T...AG.TCCTAAC	.C.G..T..A

The variable sites (1–133) in nrITS sequence (689 bp)：26, 30, 33, 34, 35, 37, 38, 45, 46, 51, 53, 56, 69, 70, 80, 82, 83, 84, 86, 88, 89, 93, 101, 106, 109, 114, 115, 116, 117, 126, 128, 139, 142, 143, 147, 148, 150, 163, 165, 167, 169, 181, 192, 193, 197, 198, 203, 207, 212, 221, 233, 234, 235, 238, 239, 257, 259, 261, 298, 314, 416, 429, 430, 435, 439, 441, 442, 443, 444, 446, 448, 452, 454, 455, 456, 458, 461, 463, 465, 467, 468, 469, 470, 471, 482, 511, 512, 515, 519, 525, 536, 537, 546, 547, 550, 551, 555, 556, 558, 562, 566, 575, 583, 584, 585, 589, 594, 595, 597, 599, 602, 603, 605, 608, 610, 614, 615, 617, 620, 623, 627, 629, 634, 635, 643, 645, 652, 657, 658, 659, 662, 669, 674.

Table S10 Variable sites of the nrITS of *A. fasciculatum* identifying six ribotypes.

Ribotype	Variable sites			
	23	24	218	391
R47	A	G	C	A
R48	.	.	T	.
R49	T	.	.	.
R50	T	C	T	.
R51	T	C	.	.
R52	.	.	.	G

Table S11 The genetic diversity of *Cyathophora* and *A. fasciculatum* in each locus of the nSSRs dataset. The significance of HWD (deviation of Hardy-Weinberg equilibrium) was indicated by * (*P* < 0.05), and ** (*P* < 0.001); NS, not significant; ND not done due to monomorphic loci. Species abbreviation: *A. fasciculatum*, AFS; *A. mairei*, AMA; *A. cyathophorum*, ACY; *A. farreri*, AFR; *A. tetraploidum*, ATE; *A. spicatum*, ASP.

**AFS**	A_O	H_S	F_{ST}	F_{is}	H_O	H_E	HWD	Null Alleles present	**AMA**	A_O	H_S	F_{ST}	F_{is}	H_O	H_E	HWD	Null Alleles present
GM102	4	0.308	0.318	0.048	0.365	0.551	*	NO	GM102	2	0.306	0.102	–0.342	0.381	0.312	ND	NO
GM63	9	0.172	0.128	0.164	0.183	0.248	ND	NO	GM63	1	0	0.457	0.275	0	0	ND	NO
GM89	15	0.401	0.357	0.249	0.357	0.721	**	YES	GM89	9	0.34	0.378	0.484	0.286	0.671	**	YES
GM011	5	0.158	0.720	–1.00	0.374	0.638	**	NO	GM011	8	0.383	0.39	–0.203	0.238	0.694	**	NO
GM109	2	0.478	0.055	–0.806	0.87	0.505	**	NO	GM109	5	0.51	0.549	0.662	0.571	0.731	NS	NO
GM001	5	0.545	0.166	–0.688	0.948	0.668	*	NO	GM001	6	0.357	0.029	0.355	0.119	0.709	**	YES
GM46	2	0.48	0.053	–0.482	0.704	0.501	**	NO	GM46	4	0.506	–0.016	0.471	0.381	0.602	NS	NO
eSSR6	6	0.148	0.697	0.674	0.061	0.59	**	YES	eSSR6	5	0.349	0.218	–0.355	0.191	0.353	ND	NO
GM112	7	0.115	0.233	0.344	0.096	0.211	ND	NO	GM112	2	0.216	0.218	–0.355	0.429	0.391	ND	NO
Mean	6.1	0.311	0.303	0.296	0.440	0.515	-	-		4.7	0.330	0.293	0.4494	0.288	0.496		
**ACY**									**AFR**								
GM102	9	0.394	0.152	–0.231	0.503	0.477	NS	NO	GM102	3	0.111	0.319	–0.437	0.086	0.084	ND	NO
GM63	10	0.067	0.02	0.025	0.075	0.079	ND	NO	GM63	7	0.126	–0.006	0.428	0.086	0.149	ND	NO
GM89	10	0.632	0.111	–0.172	0.739	0.705	*	NO	GM89	4	0.109	0.053	–0.091	0.121	0.116	ND	NO
GM011	9	0.233	0.574	0.323	0.146	0.489	**	YES	GM011	6	0.448	0.107	–0.498	0.655	0.484	NS	NO
GM109	6	0.269	0.469	0.013	0.302	0.561	**	YES	GM109	3	0.336	0.241	–0.471	0.483	0.418	ND	NO
GM001	5	0.105	0.206	–0.281	0.151	0.147	ND	NO	GM001	6	0.269	0.445	0.619	0.121	0.531	**	YES
GM46	2	0.429	0.156	–0.348	0.568	0.497	NS	NO	GM46	4	0.519	0.016	–0.608	0.845	0.535	**	NO
eSSR6	5	0.234	0.575	0.504	0.121	0.55	**	YES	eSSR6	2	0.029	–0.028	1	0	0.034	ND	NO
GM112	11	0.238	0.073	0.505	0.121	0.253	ND	NO	GM112	3	0.36	0.329	0.29	0.31	0.618	*	YES
Mean	7.4	0.289	0.260	0.274	0.302	0.418	-	-		4.2	0.256	0.216	0.584	0.301	0.330	-	-
**ASP**									**ATE**								

GM102	4	0.486	0.073	–0.191	0.581	0.524	NS	NO	GM102	3	0.048	0.027	–0.036	0.053	0.052	ND	NO
GM63	6	0.141	0.024	0.544	0.076	0.171	ND	NO	GM63	2	0.083	–0.041	0.801	0.018	0.085	ND	NO
GM89	12	0.611	0.228	–0.309	0.819	0.795	NS	NO	GM89	1	0	0.611	0.193	0	0	ND	NO
GM011	12	0.415	0.395	0.532	0.2	0.681	**	YES	GM011	9	0.254	0.127	–0.655	0.211	0.62	**	YES
GM109	2	0.193	0.226	0.667	0.067	0.253	ND	NO	GM109	2	0.372	0.096	0.068	0.649	0.442	*	NO
GM001	9	0.351	0.25	0.258	0.286	0.502	**	NO	GM001	4	0.338	0.1855	-0.188	0.456	0.369	NS	NO
GM46	4	0.422	0.123	–0.254	0.571	0.515	NS	NO	GM46	3	0.361	–0.009	0.003	0.351	0.509	NS	NO
eSSR6	5	0.366	0.187	0.69	0.133	0.52	**	YES	eSSR6	3	0.033	–0.029	1	0.035	0.035	ND	NO
GM112	11	0.602	0.225	0.402	0.381	0.806	**	YES	GM112	4	0.238	0.5115	0.92	0.018	0.462	ND	NO
Mean	7.2	0.340	0.192	0.516	0.346	0.806	-	-		3.4	0.192	0.260	0.400	0.199	0.286	-	-

Table S12 The principle complements and bioclimatic variables with significant differences tested by the PERMANOVA and Tukey's HSD tests in R packages among and within group of interest. The significance was indicated by $P < 0.05$. Species abbreviation: *A. fasciculatum*, AFS; *A. mairei*, AMA; *A. cyathophorum*, ACY; *A. farreri*, AFR; *A. tetraploidum*, ATE; *A. spicatum*, ASP.

taxa	**Bio1**	**Bio2**	**Bio3**	**Bio4**	**Bio12**	**Bio14**	**Bio15**	**Bio17**	**Altitude**	**PC1**	**PC2**
**PERM-ANOVA tests**											
East vs. West group of six species	**<0.001**	**<0.001**	0.6667	**0.0247**	-	**<0.001**	**<0.001**	-	**<0.001**	0.055	0.0961
East vs. West group of subgenus *Cyathophora*	**<0.001**	**<0.001**	0.9804	-	-	-	**<0.001**	**<0.001**	**<0.001**	0.902	0.554
East vs. West group of AFS	**0.0142**	0.548	0.0927	0.0913	**<0.001**	**0.0134**	**0.0096**	-	**<0.001**	0.204	0.253
East-AFS vs. ACY	0.5412	0.9804	0.2025	0.0767	0.3533	0.7255	0.3317	-	0.2304	0.3657	0.5000
West-AFS vs. ASP	0.1190	-	0.8627	0.8039	0.4906	0.9412	0.6333	-	0.0760	0.863	0.2910
**Tukey's HSD tests**											
East vs. West group of six species	**<0.001**	**<0.001**	0.540	**0.0251**	-	**<0.001**	**<0.001**	-	**<0.001**	**0.0137**	0.4339
East vs. West group of subgenus *Cyathophora*	**0.0005**	**0.0006**	0.7293	-	-	-	**<0.001**	**0.0008**	**<0.001**	0.9203	0.6234
East vs. West group of AFS	**0.0123**	0.5631	0.1115	0.1680	**<0.001**	**0.0032**	**0.0039**	-	**0.0005**	0.2030	0.2459
East-AFS vs. ACY	0.9887	0.9903	0.9553	0.7964	-	-	0.9413	-	0.9759	0.9835	0.9992
West-AFS vs. ASP	0.1330	-	0.9065	0.8421	0.5887	0.6984	0.6533	-	0.0984	0.6224	0.3488

Table S13 The mantel tests between pairwise $F_{ST}/(1 - F_{ST})$ of nSSRs and each determinant bioclimatic variable and principle components (PC1 and PC2).

**Groups**	**Bioclimatic variable**							
**nSSRs**	Bio1	Bio2	Bio3	Bio12	Bio14	Bio15	Altitude	Bio17
**east-west groups of A. fasciculatum**	r=0.1628 *P*=0.07	-	-	r=0.1282 *P*=0.088	r=0.0173 *P*=0.331	r=0.1828 *P*=0.061	r=0.1528 *P*=0.083	-
**East-west groups of subgenus Cyathophora**	r=0.0273 *P*=0.354	r=0.132 *P*=**0.007**	r=0.1555 *P*=**0.012**	-	r=0.0173 *P*=0.331	r=0.1237 *P*=0.057	r=0.0725 *P*=0.15	r= –0.224 *P*=0.595
